# Gli1 Deletion Prevents *Helicobacter*-Induced Gastric Metaplasia and Expansion of Myeloid Cell Subsets

**DOI:** 10.1371/journal.pone.0058935

**Published:** 2013-03-08

**Authors:** Mohamad El-Zaatari, John Y. Kao, Art Tessier, Longchuan Bai, Michael M. Hayes, Clinton Fontaine, Kathryn A. Eaton, Juanita L. Merchant

**Affiliations:** 1 Department of Internal Medicine, Division of Gastroenterology, University of Michigan, Ann Arbor, Michigan, United States of America; 2 Department of Microbiology and Immunology, University of Michigan, Ann Arbor, Michigan, United States of America; Vanderbilt University Medical Center, United States of America

## Abstract

Chronic inflammation in the stomach induces metaplasia, the pre-cancerous lesion that precedes inflammation-driven neoplastic transformation. While Hedgehog signaling contributes to the initiation of some cancers, its role in gastric transformation remains poorly defined. We found that *Helicobacter*-infected C57BL/6 mice develop extensive mucous cell metaplasia at 6 month but not at 2 months post-infection. Gastric metaplasia coincided with the appearance of CD45^+^MHCII^+^CD11b^+^CD11c^+^ myeloid cells that were normally not present in the chronic gastritis at 2 months. The myeloid regulatory gene *Schlafen-4* was identified in a microarray analysis comparing infected WT versus *Gli1* null mice and was expressed in the CD11b^+^CD11c^+^ myeloid population. Moreover this same population expressed IL-1β and TNFα pro-inflammatory cytokines. By 6 months, the mucous neck cell metaplasia (SPEM) expressed IL-6, phosphorylated STAT3 and the proliferative marker Ki67. Expression was not observed in *Gli1* mutant mice consistent with the requirement of *Gli1* to induce this pre-neoplastic phenotype. Ectopic Shh ligand expression alone was not sufficient to induce SPEM, but with *Helicobacter* infection synergistically increased the histologic severity observed with the inflammation. Therefore Hedgehog signaling is required, but is not sufficient to generate pre-neoplastic changes during chronic gastritis. Gli1-dependent myeloid cell differentiation plays a pivotal role in the appearance of myeloid cell subtypes ostensibly required for SPEM development. Moreover, it suggests that therapies capable of targeting this phenotypic switch might prevent progression to metaplasia, the pre-neoplastic change that develops prior to dysplasia and gastric cancer, which also occurs in other epithelial-derived neoplasias initiated by chronic inflammation.

## Introduction

Gastric metaplasia is the histologic change that precedes neoplastic transformation of the stomach in response to inflammation [1]. The gastric mucosa is primarily composed of acid-producing (parietal cells), pepsinogen-producing (chief cells), and mucus-producing (surface pit and neck) cells [2]. During *Helicobacter pylori* (*H. pylori*) infection, the gastric epithelium expands mucous cell compartments at the expense of parietal and chief cells, thereby reducing acid secretion [3]. Cytokine-secreting myeloid cells are a component of the bacteria-induced inflammation infiltrating the gastric stromal microenvironment [4]. Over time, these pro-inflammatory cytokines are sufficient to initiate pre-neoplastic changes in the epithelium [5].

Aberrant Hedgehog (Hh) signaling promotes the development of medulloblastoma, basal cell, pancreatic and colon carcinoma [6]. However, its role in gastric cancer development is less clear. We and others previously established that gastric parietal cells produce Sonic Hedgehog (Shh) ligand [7], [8], [9], [10], which activates one of several effector genes including the glioma-associated oncogene 1 (Gli1) transcription factor expressed in cells residing in the lamina propria such as immune cells and myofibroblasts [11]. A recent report showed that Hh signaling is necessary for myeloid cell recruitment to the stomach within two days of *H. pylori* infection [12], but the downstream effects of the Hh pathway leading to pre-neoplastic transformation were not examined. Therefore to test whether Hh signaling is required for gastric transformation, we infected wild type C57BL/6 (WT) and *Gli1*-deficient mice with *Helicobacter felis* (*H. felis*) to generate a robust inflammatory response (chronic gastritis) and gastric metaplasia.

## Materials and Methods

### Transgenic Mice

C57BL/6 and Gli1^+/LacZ^ (Gli1^+/−^) mice were obtained from Jackson Labs (Bar Harbor, ME) and bred to generate Gli1^LacZ/LacZ^ (Gli1^−/−^) mice. These mice contain a knock-in of the cDNA for nuclear **β**-galactosidase (nLacZ) inserted into exon 2 of the Gli1 gene abolishing endogenous gene expression. Transgenic founders were generated by micro-injecting CMV-Shh^WT-HA^, CMV-Shh^F200H-HA^, H^+^/K^+^-ATPase-**β**-Shh^WT-HA^ and H^+^/K^+^-ATPase-**β**-Shh^F200H-HA^ constructs into fertilized eggs obtained by mating (C57BL/6/J X SJL/J)F1 males and females of the same background (UM Transgenic Core, Ann Arbor, MI). The N-terminal Hemagglutinin (HA)-tagged mouse Shh constructs were generated by inserting four copies of the HA epitope (TACCCTTACGACGTTCCTGATTACGCT) downstream of nucleotide 96 (amino acid 32) in the Shh cDNA sequence. The F200H mutant Shh transgene was generated by substituting “TT” nucleotides for “CA” at position 597 of the mouse Shh cDNA using the QuickChange site-directed mutagenesis kit (Stratagene, Santa Clara, CA). These sequences were subcloned into the topoisomerase site of pcDNA3.1/V5-His-TOPO and into the BamHI restriction site of the mouse H^+^/K^+^-ATPase-β subunit expression plasmid (–1,035 to +24). Mice were housed under specific pathogen-free conditions and fasted overnight prior to use, with free access to water. All mouse studies were approved by the University of Michigan Institutional Animal Care and Use Committee.

### *Helicobacter felis* culture and infection

*H. felis* (CS1 strain) stocks were stored in 50% glycerol solution at −80°C. Bacteria were cultured in sterile-filtered Brucella broth (BD, Franklin Lakes, NJ) plus 10% FBS (Atlanta Biologicals, Lawrenceville, GA) using the GasPak™ EZ Campy Container System (BD) at 37°C with 150 rpm shaking. The cultures were spun down at 2700 rpm at room temperature, and the pellets resuspended in Brucella broth plus 10% FBS (Thermo Fisher Scientific, Houston, TX). Cells were counted using a hemocytometer by diluting the cells 1∶100 in 9∶1 HBSS/Formalin solution. Mice were gavaged 3 times over 3 days with 10^8^ *H. felis* cells in 100 μL of Brucella broth. Control mice were gavaged with Brucella broth alone.

### *H. felis* DNA quantification

Gastric tissue from the corpus and fundus was snap frozen and stored at −80°C. Total DNA was extracted using the DNEasy Blood and Tissue Kit (Qiagen). Quantitative PCR was performed using the *H. felis* Fla-B primers-F: 5′TTCGATTGGTCCTACAGGCTCAGA, R: 5′TTCTTGTTGATGACATTGACCAACGCA 3′ on a CFX96 real-time PCR detection system (Bio-RAD).

### Tissue Preparation

Mice were starved overnight then euthanized. The stomachs were removed, opened along the greater curvature, and cut into longitudinal strips for histology from the lesser and greater curvatures. Half of the strips were fixed in 4% formaldehyde (Fisher Scientific) and the other half directly embedded in OCT compound (Fisher Scientific) and snap-frozen. The remainder of the stomach, containing only fundus and corpus, was minced and processed for RNA extraction or digested for flow cytometric analysis.

### Immunofluorescence

For frozen sections, 8 µm sections were fixed in 4% paraformaldehyde for 10 min, washed in PBS twice, and then blocked with 20% donkey serum (#017-000-121, Jackson ImmunoResearch, West Grove, PA) in PBS. Frozen sections were immunostained with the following antibodies: **β**-gal (gift from James Douglas Engel, Department of Cell and Developmental Biology, University of Michigan), TFF-2 (gift from Nicholas Wright, Barts and The London School of Medicine, London, UK), F4/80 (#MCA497GA, AbD Serotec, Raleigh, NC), CD11b (#ab6332-100, clone M1/70.15, Abcam, Cambridge, MA), CD11c-FITC (#553801, BD Pharmingen, BD Bioscience, Bedford, MA), α-SMA-Cy3 (#C6198, Sigma, St Louis, MO), CD19 (#MCA1439, AbD Serotec), MPO-FITC (#90812, Abcam), Slfn-4 (#sc-8903, Santa Cruz Biotechnology, Santa Cruz, CA), pSTAT-3 (#9131, Cell Signaling, Boston, MA), IL-1β (#AF-401-NA, R&D Systems, Minneapolis, MN), Ki-67 (#RM-9106-S1, Thermo Scientific, Fisher), Shh (#sc-1194, Santa Cruz, CA), E-cadherin (#3195, Cell Signaling), and GSII-FITC (#FL-1211, Vector Labs, Burlingame, CA). For unlabeled primary antibodies, the staining was developed using Alexa Fluor-conjugated secondary antibodies (Molecular Probes, Invitrogen, Carlsbad, CA).

For paraffin sections, 8 µm sections were deparaffinized in xylene and 100% ethanol. Sections were re-hydrated with distilled water and antigen retrieval was performed using 10 mM citric acid buffer (pH 6). Slides were washed in 0.01% Triton X-100 (Fisher) in PBS twice, incubated with 20% donkey serum (#017-000-121, Jackson ImmunoResearch) and immunostained with the following antibodies: H^+^/K^+^-ATPase-β (#D032-3, Medical and Biological Laboratories, Woburn, MA), HA-probe (#sc-7392, Santa Cruz), Shh (#sc-1194, Santa Cruz), intrinsic factor (gift from David Alpers, Washington University, St. Louis, MO), and GSII-FITC (#FL-1211, Vector Labs). For unlabeled primary antibodies, staining was developed using Alexa Fluor-conjugated secondary antibodies (Molecular Probes, Invitrogen). Concentration and species matched immunoglobulins were used as controls for each antibody. Staining was visualized using an Olympus Fluoview scanning confocal microscope (Olympus, Center Valley, PA).

### Morphometric Analysis

Well-oriented gastric glands from the fundus and corpus were observed under a 20× objective lens (200× total magnification) and scored for neutrophilic infiltration (polymorphonuclear leukocytes [PMN]), gastritis, follicle formation, and metaplasia. Each microscopic field was scored only for the presence or absence of the lesion according to the following criteria: 1) neutrophilic inflammation was defined by the presence of several clusters of neutrophils within the mucosa; 2) “displacement of epithelial glands” is defined as the displacement of gastric glands by inflammatory cell infiltrates of any cell type; 3) metaplasia was defined by the presence of an expansion of the mucous cell lineage compartment. For each slide, the number of positive fields was divided by the total number of fields examined and was expressed as a percentage of the affected fields.

### Real-time quantitative PCR

The forestomach was removed and tissue from the glandular stomach was minced and homogenized in TRIzol (Invitrogen). RNA clean-up was performed using the RNeasy Minikit (Qiagen, Valencia, CA), and cDNA synthesized from 1 µg of RNA using iScript reverse transcriptase (Bio-Rad, Hercules, CA). Real-time quantitative polymerase chain reaction (RT-qPCR) was performed using Platinum Taq DNA polymerase (Invitrogen) on a CFX96 real-time PCR detection system (Bio-RAD), using the following primer sequences (Tm  = 65°C for all primers):

CD11b-F: 5′CTCCGGTAGCATCAACAACAT, R: 5^′^TGATCTTGGGCTAGGGTTTCT;

CD11c-F: 5′CTGGATAGCCTTTCTTCTGCTG, R: 5′GCACACTGTGTCCGAACTC;

F4/80-F: 5′CCCAGCTTCTGCCACCTGCA, R: 5′GGAGCCATTCAAGACAAAGCC;

Gli1-F: 5′TTGGGATGAAGAAGCAGTTG, R: 5′GGAGACAGCATGGCTCACTA;

HPRT-F: 5′AGGACCTCTCGAAGTGTTGGATAC, R: 5′AACTTGCGCTCATCTTAGGCTTTG;

IFN-γ-F: 5′TCAAGTGGCATAGATGTGGAAGAA, R: 5′TGGCTCTGCAGGATTTTCATG;

IL-12p35-F: 5′AAATGAAGCTCTGCATCCTGC, R: 5′TCACCCTGTTGATGGTCACG;

IL-12p40-F: 5′AAACCAGACCCGCCCAAGAAC, R 5′AAAAAGCCAACCAAGCAGAAGACAG;

IL-4 F: 5′ACAGGAGAAGGGACGCCAT, R: 5′GAAGCCCTACAGACGAGCTCA;

IL-5-F: 5′AGCACAGTGGTGAAAGAGACCTT, R: 5′TCCAATGCATAGCTGGTGATTT,

IL-6-F: 5′GAGGATACCACTCCCAACAGACC, R: 5′AAGTGCATCATCGTTGTTCATACA;

IL1-β-F: 5′CAACCAACAAGTGATATTCTCCATG, R: 5′GATCCACACTCTCCAGCTGCA;

IL17A-F: 5′GCTCCAGAAGGCCCTCAGA, R: 5′AGCTTTCCCTCCGCATTGA;

KC-F: 5′CTGCACCCAAACCGAAGTCAT, R: 5′TTGTCAGAAGCCAGCGTTCAC;

MPO-F: 5′CCAGCAGCCATGAAGTA, R: 5′CATAACGGAAAGCATTGGTG;

Ptch1-F: 5′CTGCTGTGGTGGTGGTATTC, R: 5′GGCTTGTGAAACAGCAGAAA;

SAA3-F: 5′TGCCATCATTCTTTGCATCTTGA, R: 5′CCGTGAACTTCTGAACAGCCT;

Shh-F: 5′ATGTTTTCTGGTGATCCTTGCT, R: 5′ATCGTTCGGAGTTTCTTGTGAT;

Slfn-4-F: 5′GCCCTCTGTTCAAGTCAAGTGTCC, R: 5′CCCAGATGAAATCCTTTCCACGA;

TNF-α-F: 5′CATCTTCTCAAAATTCGAGTGACAA, R: 5′TGGGAGTAGACAAGGTACAACCC;

CCL-19-F: 5′TGTGGCCTGCCTCAG ATTAT, R: 5′AGTCTTCCGCATCATTAGCAC;

CD3-F: 5′TGCTCTTGGTGTATATCTC ATTGC, R: 5′CAGAGTCTGCTTGTCTGAAGCTC.

### Microarray analysis

Microarray analysis was performed by the Microarray Core Facility at the University of Michigan. Briefly, RNA was labeled by preparing biotinylated antisense RNA (aRNA) from 250 ng of total RNA according to the Affymetrix GeneChip 3′ IVT Express kit protocol (Affymetrix, Santa Clara, CA). Following fragmentation, 7.5 µg of aRNA were hybridized for 16 hr at 45°C to the Mouse Genome 430 2.0 Perfect Match Peg Arrays (Affymetrix) and scanned using the Affymetrix Gene Atlas system (software version 1.0.4.267, Affymetrix).

### Laser Capture Microdissection

Frozen sections (10 µm) were mounted on Leica Pen-Membrane (2 µm) slides (Leica Microsystems Inc., Buffalo Grove, IL) and stored at −20°C until further use. Frozen sections were air-dried, fixed in RNase-free 70% ethanol (pre-chilled, −30°C) for 5 min at room temperature, air-dried, stained in 0.25% RNase-free toluidine blue for 10 sec, then washed in 70% ethanol (pre-chilled, −30°C). Sections were air-dried and the microdissectates collected using a Leica LMD 7000 microdissection microscope (Leica Microsystems Inc.).

### Flow cytometry

Stomach tissue was minced, incubated in 10 ml HBSS supplemented with 1 mM DTT (Sigma) and 1 mM EDTA (#51201 Lonza, NJ, USA), and then shaken at 160 rpm for 1 hr at 37°C. The supernatant was collected by passing the 10 ml sample through a 40 µm cell strainer (#08-771-1 Fisher). The tissue collected in the cell strainer was further digested in 10 ml of RPMI (without L-glutamine) containing 5% BSA (#9048-46-8 Roche) and 1.5 mg/ml Dispase II (#04942078001 Roche) with 160 rpm shaking at 37°C for 90 min. The supernatant was collected by passing the digested tissue through a 40 µm cell strainer and both supernatants pooled. For bone marrow cells, the femur marrow was directly flushed with RPMI and treated with ACK lysis buffer (#10-548E Lonza) to lyse red blood cells. The cells were spun down at 1600 rpm, resuspended in 200 µl of PBS containing 5% BSA (Roche), blocked with purified rat anti-mouse DC16/CD32 mouse Fc block (#553141 BD Biosciences, San Diego, CA) at 4°C for 5 min, and incubated with F4/80-FITC (#MCA497FT AbD Serotec), CD11b-PE-Cy7 (#561098 BD Pharmingen, BD Biosciences), CD11c-APC (#561119 BD Biosciences), CD45-PE (#12-0451-81 eBioscience), pSTAT3 (pY705)-PerCP-Cy5.5 (#560114 BD Biosciences), EpCAM-APC (#17-5791-80 eBioscience), GSII-FITC (#FL-1211 Vector Labs), or IL-6-PE (#562050 BD Biosciences). The cells were fixed in 100 µl of Reagent A (#GAS001S5 Invitrogen), washed, and resuspended in 500 µl PBS containing 5% BSA in a BD Falcon tube (#352052 BD). For pSTAT3-PerCP-Cy5.5 an additional permeabilization step was added by incubating the cells in Reagent B (#GAS002S5 Invitrogen). The cells were analyzed using an Accuri C6 Flow Cytometer (Accuri Cytometers, Ann Arbor, MI).

To flow sort cells for subsequent RT-PCR analysis, gastric cells were prepared as described above and gated for CD45, F4/80 and CD11c-expressing cells using the CD45-PE (#12-0451-81 eBioscience), F4/80-FITC (#MCA497FT AbD Serotec) and CD11c-APC (#561119 BD Biosciences) antibodies. Cells were collected in buffer RLT containing β-mercaptoethanol and carrier RNA using a sy3200 (Synergy) sorter (iCyt, Champaign, IL) then extracted using the RNEasy Microkit (Qiagen, Valencia, CA) according to the manufacturer's instructions.

### Cell Culture Experiments

NCI-N87 mucous cells (#CRL-5822 ATCC) were plated in 24 well plates, serum-starved overnight, and then treated with recombinant human IL-6 (100 ng/ml, 206-IL-010/CF, R&D Systems) or IL-1β (1 ng/ml, 201-LB-005, R&D Systems) overnight. IL-1RA (10 ng/ml) was added 2 hrs prior to IL-1β treatment. The cells were lysed in TRIzol for RNA extraction and RT-qPCR analysis.

### Western Blot Analysis

The cell lysates of NIH-3T3 cells were homogenized in T-per extraction reagent (78510, Pierce, Rockford, IL). The protein lysate (100 µg) was resolved on a Novex 4–20% Tris-Glycine gel (#EC6025 Invitrogen) and then transferred to nitrocellulose using the iBlot Dry Blotting System (Invitrogen) according to the manufacturer's instructions. The membranes were incubated in KPL Detector Block (#71-83-00 Gaithersburg, MD) for 1 hour at room temperature and then incubated with 1∶100 Shh (#sc-1194; Santa Cruz) or 1∶1000 HA-tag (#sc-7392, Santa Cruz) antibodies at 4°C overnight. Membranes were washed with TBS containing 0.1% Tween, incubated with IRDye 680-conjugated antibodies (Licor, Lincoln, NB), and visualized with an Odyssey infrared imager (Licor).

### Chromatin Immunoprecipitation (ChIP) assay

Gli1 binding sites in the *Slfn-4*, *Slfn-2* and *SAA3* promoters were identified using Genomatix (Munich, Germany). Primers were designed to amplify the nucleotide sequence between −3323 and −3101 of the Slfn4 promoter, which contains a GGCCACCCA Gli1 binding site (consensus sequence GACCACCCA). The primers were designed as follows: F 5′ AAGGCAGAAACATG GCAGCATCC 3′; R 5′ ACTTTGGCTTGTGTCAGGTTGGC 3′. A nucleotide sequence between −589 and −385 that lacks a Gli1 binding site was used as a negative control and was amplified using the following primers: F 5′ TGTGTTCCTGAGTGTGAGTGTTC 3′; R 5′ CTTCCCAGAGCAG CCTTAGC 3′. Flag-tagged pcDNA3.1-Gli1 (gift from Andrzej Dlugosz, Department of Dermatology, University of Michigan) or empty vector were transfected into RAW264.7 cells. ChIP was performed using the Flag-tag antibody (#2368 Cell Signaling) and the Chromatin Immunoprecipitation (ChIP) Assay Kit (#17–295 Millipore, Kankakee, IL).

### Statistical Analysis

For *in vivo* qPCR analysis and pathological scoring of mice, each bar graph represented the mean +/− standard error from 5–10 mice. For cell culture experiments, error bars represented the means +/− standard error of the mean from 3 independent experiments. Data were tested for normality using the Shapiro-Wilk W test (Prism, GraphPad Software, La Jolla, CA). Data were compared using one-way analysis of variance (ANOVA) with Dunnet's (parametric) or Dunn's (non-parametric) multiple comparison tests (Prism). *P* values less than 0.05 were considered significant.

### Microarray Accession Number

The data discussed in this publication have been deposited in NCBI's Gene Expression Omnibus (El-Zaatari et al., 2013) and are accessible through GEO Series accession number GSE43693 (http://www.ncbi.nlm.nih.gov/geo/query/acc.cgi?acc=GSE43693).

## Results

### *Gli1* Deletion Prevents Gastric Metaplasia

**Figure 1:**
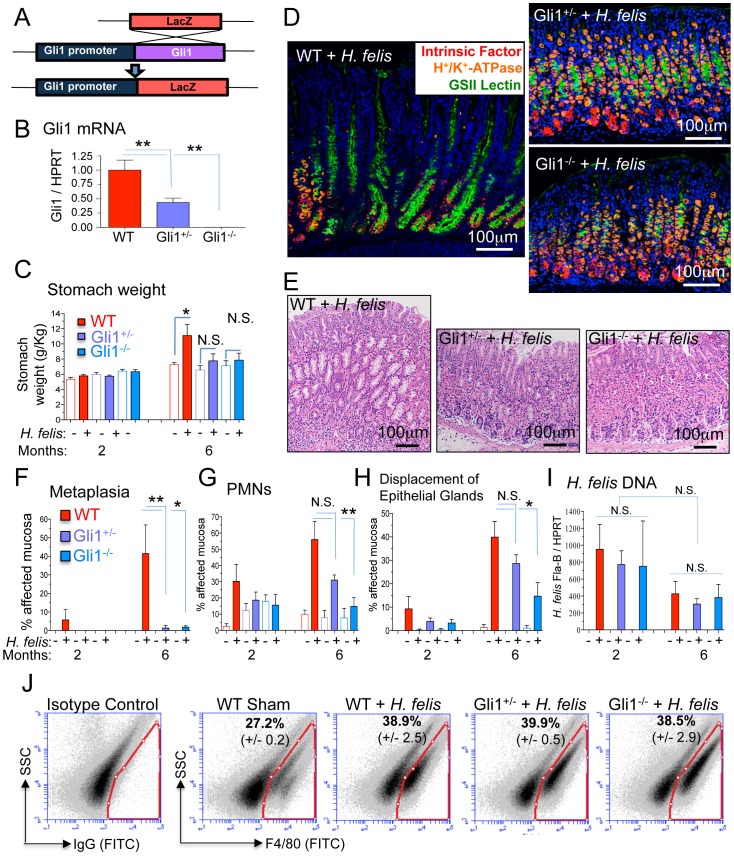
*Gli1* deletion prevents gastric metaplasia. A) Schematic representation of LacZ substitution for the Gli1 gene to generate Gli1^+/−^ and Gli1^−/−^ mice. B) Gli1 mRNA in stomachs of WT, Gli1^+/−^ and Gli1^−/−^ mice. C) Stomach weight normalized to total body weight in infected and uninfected mice. D) Triple immunofluorescent staining of *Griffonia simplicifolia* II (GSII) lectin (green; mucous neck cells), intrinsic factor (red; chief cells), and H^+^/K^+^-ATPase (orange; parietal cells) in 6-month *H. felis*-infected WT, Gli1^+/−^ and Gli1^−/−^ stomachs. E) Hematoxylin and eosin (H&E) staining of the gastric mucosa in 6-month *H. felis*-infected WT, Gli1^+/−^ and Gli1^−/−^. F–H) Histologic scoring of metaplasia, PMN infiltration, and displacement of epithelial glands by infiltrating inflammatory cells in 2- and 6-month *H. felis*-infected WT, Gli1^+/−^ and Gli1^−/−^ stomachs. I) qPCR quantification of *H. felis* flagellar filament B (Fla-B) DNA in the infected mucosa. J) Flow cytometric analysis of F4/80^+^ cells versus side-scatter in WT Sham and 6-month infected WT, Gli1^+/−^ and Gli1^−/−^ stomachs (N = 3 mice per group). Open and closed bars denote uninfected and infected mice respectively. For RT-qPCR experiments, N = 5–10 mice per group. Error bars represent the mean +/− SEM. ***p<0.001; **p<0.01; *p<0.05. N.S.  =  not significant.

To understand the role of Hedgehog signaling in gastric metaplasia, WT, Gli1^+/LacZ^ (Gli1^+/−^) and Gli1^LacZ/LacZ^ (Gli1^−/−^) mice (Figure 1A) were infected with *H. felis*. Gli1^+/−^ and Gli1^−/−^ mice expressed significantly reduced or absent *Gli1* mRNA in the stomach respectively (Figure 1B). Although inflammation was observed 2 months after inoculating the WT mice, by 6 months there was an increase in stomach weight (Figure 1C), loss of parietal and chief cells, and development of spasmolytic polypeptide-expressing metaplasia (SPEM) characterized by the expansion of the mucous neck cell compartment (Figure 1D and E) and co-labeling of mucous cells with the chief cell lineage marker intrinsic factor (Figure S1) [3]. The amount of SPEM (Figure 1F and Figure S1), polymorphic neutrophil (PMN) infiltration (Figure 1G) and epithelial gland displacement (Figure 1H) was greatest in the infected WT mice. In contrast, Gli1^+/−^ and Gli1^−/−^ mice were also inflamed but lacked any SPEM (Figure 1D-F). These differences were not due to differences in *H. felis* colonization between WT, Gli1^+/−^ or Gli1^−/−^ mice (Figure 1I). Despite the absence of metaplasia, there were similar amounts of F4/80^+^ myeloid cells infiltrating the infected WT, Gli1^+/−^ and Gli1^−/−^ stomachs (Figure 1J), demonstrating that Gli1 deletion prevented the transition from gastritis to SPEM.

### *Gli1* Deletion Prevents Th1, Th2, not Th17 inflammatory responses

**Figure 2:**
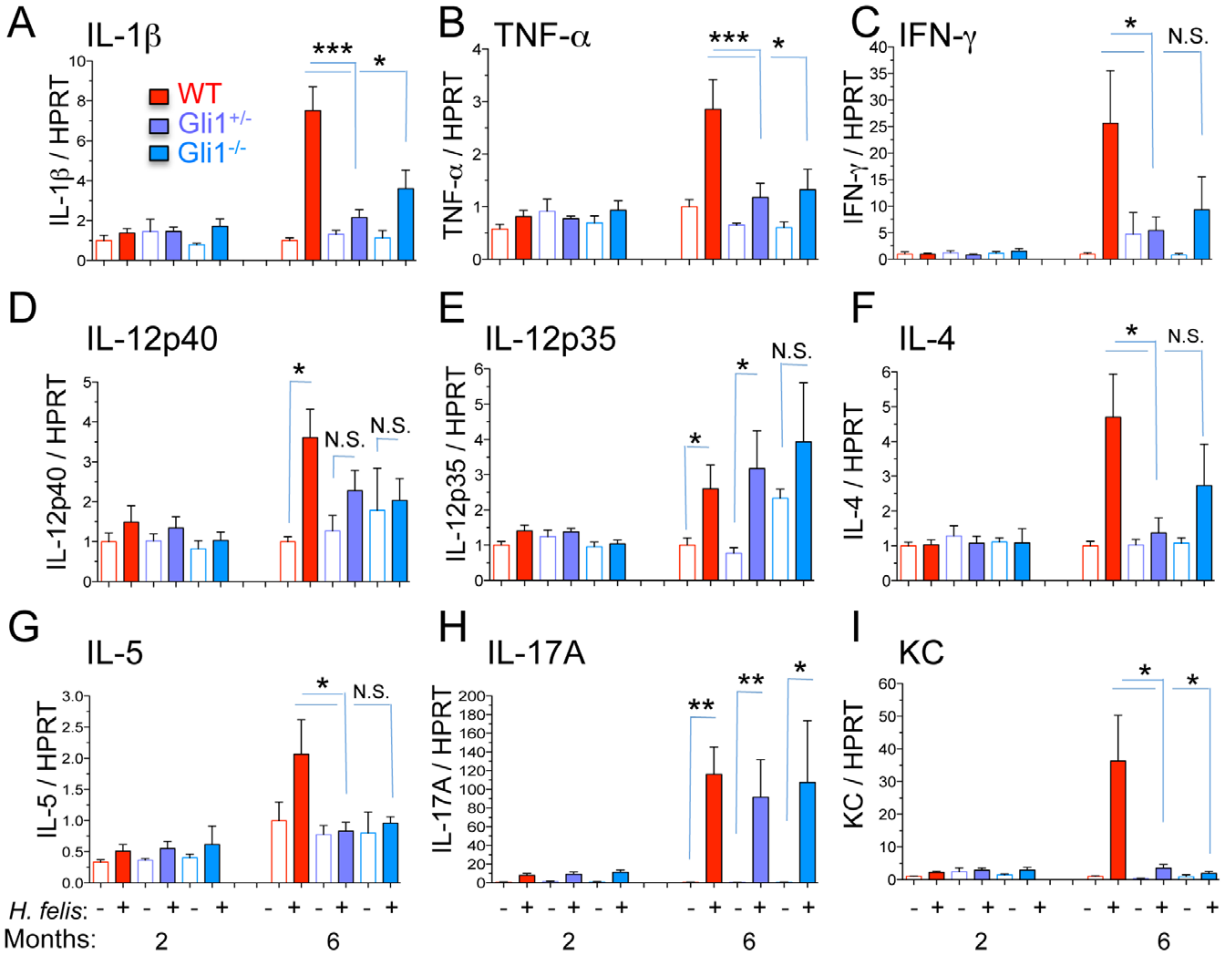
*Gli1* deletion blocks increase in Th1 and Th2 cytokines. A–E) RT-qPCR determination of mRNA for Th1-associated cytokines. F–G) RT-qPCR of mRNA for Th2-associated cytokines. H) RT-qPCR of mRNA for the Th17-associated cytokine IL-17A. I) RT-qPCR analysis of the mRNA for the mouse IL-8 ortholog, chemokine KC. Open and closed bars denote uninfected and infected mice respectively. N = 5–10 mice per group. Error bars represent the mean +/− SEM. **p<0.01; *p<0.05.

*H. felis* normally induces a complex inflammatory response in the stomach including increased Th1, Th2 and Th17 T helper lymphocyte subsets [13], [14], [15]. By 6-months, *H. felis* infection of WT mice induced the gene expression of Th1 (Figure 2 A–E), Th2 (Figure 2 F and G), and Th17 cytokines (Figure 2H) in addition to the chemokine KC (Figure 2I), the mouse IL-8 ortholog. In contrast, *H. felis* failed to induce several of these cytokines in the Gli1^+/−^ and Gli1^−/−^ stomachs except for the p35 subunit of IL-12 (Figure 2E) and IL-17A (Figure 2H), suggesting that these cytokines were not affected by loss of Gli1 and did not significantly contribute to the development of SPEM.

### Gastric Myeloid Cells Express *Gli1*

**Figure 3:**
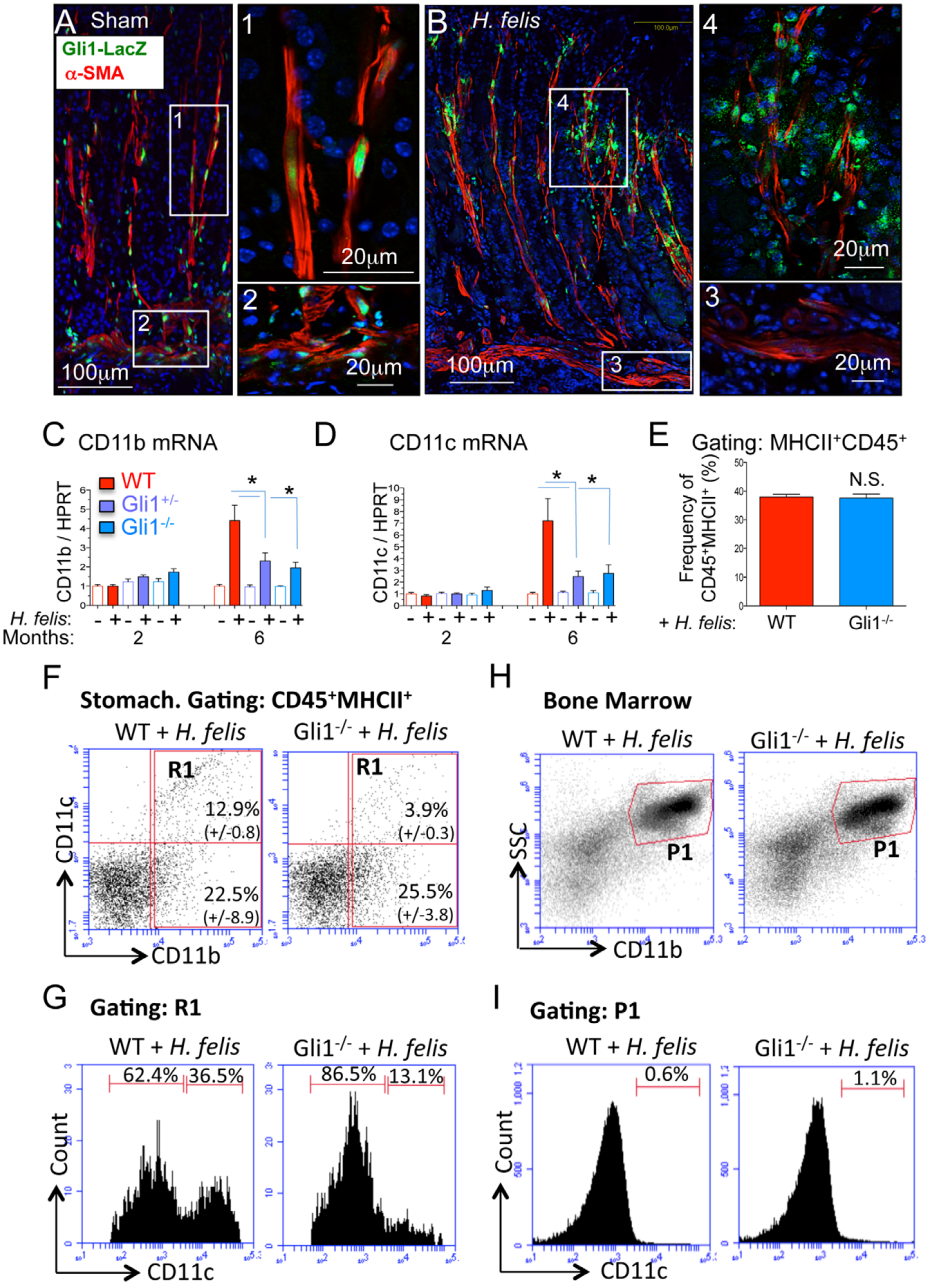
*Gli1* deletion prevents expansion of CD11b^+^CD11c^+^ myeloid cell subsets. A–B) Immunofluorescent detection of β-gal (green), α-SMA (red), and DAPI (blue) in sham- and *H. felis*-infected Gli1^+/LacZ^ mice. C–D) RT-qPCR analysis of CD11b and CD11c in 2- and 6-month infected mice. E) Bar graphs representing the percentages of CD45^+^MHCII^+^ myeloid cells per total gastric cell number in 6-month *H. felis*-infected WT versus Gli1^−/−^ mice. F) Flow cytometric analysis of CD11b and CD11c in 6-month infected WT and Gli1^−/−^ mice. Values on dot plot represent the percentages of cells (relative to total CD45^+^MHCII^+^ myeloid cell population) +/− SEM from N = 3 mice per group. G) Flow cytometry of CD11c+ cells within the CD11b^+^ stomach population (R1 gate from Figure 3F) of 6-month *H. felis*-infected WT versus Gli1^−/−^ mice. H) Flow cytometric analysis of CD11b^+^ bone marrow cells in 6-month *H. felis*-infected WT and Gli1^−/−^ mice. I) Flow cytometry of CD11c^+^ cells within the CD11b^+^ bone marrow population (P1 gate from Figure 3H) of 6-month *H. felis*-infected WT and Gli1^−/−^ mice. Open and closed bars denote uninfected and infected mice respectively. Error bars represent the mean +/− SEM. For RT-qPCR graphs, N = 5–10 mice per group. For flow sorting bar graph, N = 3 mice per group. Values on flow cytometric dot blots represent the mean +/− SEM from 3 mice per group. *p<0.05.

Gumucio and coworkers previously showed that intestinal myeloid cells and myofibroblasts express *Gli1* [11]. Myeloid cells are a major source of IL-1β [16], one of the major cytokines sufficient to induce gastric metaplasia [5], [17]. In addition, the expected induction of IL-1β gene expression was reduced in the infected *Gli1* mutant mice (Figure 2A), suggesting that *Gli1* expression contributes to myeloid cell production of 1L-1β. To characterize the immunological defect in the *Gli1* mutant mice, we identified myeloid cell subsets in the stomachs of infected WT and mutant mice. Using the LacZ reporter to identify *Gli1*-expressing cells, we found that the LacZ reporter was expressed primarily in α-SMA^+^ myofibroblasts and smooth muscle cells in the uninfected stomach as previously reported [11] (Figure 3A). However *LacZ* expression was significantly reduced in these resident mesenchymal cells 6 months after *H. felis* infection (Figure 3 A and B, *insets 1–3*), but was detected in a population of infiltrating cells that were F4/80^+^ (Figure 3B *inset 4* and Figure S2A), CD11b^+^ (Figure S2B), and CD11c^+^ (Figure S2C). *LacZ* expression was not detected in CD19^+^ (B lymphocytes, except for a few transitional cells at the follicle border, Figure S3A, *inset*), CD3^+^ (T lymphocytes, Figure S3B) or myeloperoxidase^+^ cells (MPO, PMNs, Figure S3C). No *LacZ* expression was detected with the isotype control IgGs (Figure S3D) or in WT mice lacking the *LacZ* gene insert (Figure S3E). The immunohistochemical analysis was consistent with reduced *CD11b* and *CD11c* surface marker gene expression in the *Gli1* mutant mice (Figure 3 C–D). However the total frequency of CD45^+^MHCII^+^ myeloid cells between 6-month infected WT and Gli1^−/−^ mice was not affected (Figure 3E), indicating a shift in the composition of the myeloid population rather than a defect in total myeloid cell recruitment. Indeed, flow cytometry revealed no significant difference in CD11b^+^CD11c^−^ cells but instead a 70% reduction in CD11b^+^CD11c^+^ myeloid cells in the infected Gli1^−/−^ mice (Figure 3F). This corresponded to a shift in the frequency of CD11c^+^ cells within the CD11b^+^ population from 36.5% to 13.1% (Figure 3G). Consistent with this result, we detected by immunofluorescence a significant loss of CD11c^+^, but not CD11b^+^ cells in the infected Gli1^+/−^ and Gli1^−/−^ mouse stomachs (Figure S4). Analysis of the bone marrow revealed no detectable CD11b^+^CD11c^+^ cells (Figure 3H and I). Although we cannot exclude the presence of CD11b^+^CD11c^+^ cells in the bone marrow, which might be dwarfed by the abundance of single positive cells, we concluded that CD11b^+^CD11c^−^ cells differentiate into a CD11b^+^CD11c^+^ population in the stomach in a Gli1-dependent manner. The acquisition of CD11b^+^CD11c^+^ cells in the *Helicobacter*-infected stomachs at 6 months mirrors the CD11b^+^ subset of tumor infiltrating myelomonocytoid cells described by Umemura et al. that acquire the CD11c^+^Gr1^+^ surface markers along with their T cell suppressor function (myeloid-derived suppressor function or MDSCs) [18]. Therefore we concluded that Gli1 is necessary for the differentiation of myeloid cells into CD11b^+^CD11c^+^ cells within the preneoplastic gastric microenvironment that correlates with the SPEM.

### Schlafen-4 is a Gli1-target gene that marks the CD11b^+^CD11c^+^ population associated with gastric metaplasia

**Figure 4:**
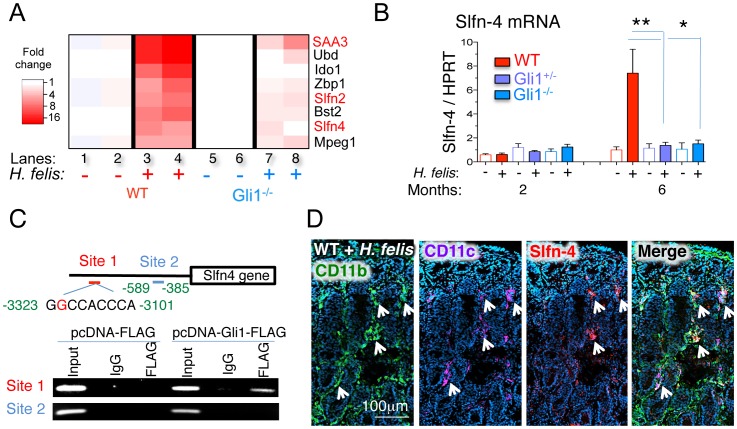
*Schlafen-4* is a Gli1-target gene that marks the CD11b^+^CD11c^+^ gastric myeloid population. A) Microarray heat map of sham- and *H. felis*-infected WT and Gli1^−/−^ stomachs. Each column represents pooled stomach RNA samples from two mice. B) RT-qPCR analysis of *Slfn-4* mRNA from sham- or 6 month *H. felis*-infected WT, Gli1^+/−^, or Gli1^−/−^ stomachs. C) ChIP analysis of two *Slfn-4* promoter sequences in mouse Gli1 promoter in RAW264.7 cells transfected with Gli1-FLAG-overexpressing or the empty vector plasmid. D) Immunofluorescence analysis of CD11b (green), CD11c (magenta), Slfn-4 (red) in 6-month *H. felis* infected WT mice. Error bars represent the mean +/− SEM. N = 5–10 mice per group. **p<0.01; *p<0.05.

To identify Gli1-dependent myeloid regulatory factors, we performed a microarray analysis of gastric tissue from *Helicobacter*-infected WT and Gli1^−/−^ mice. The genome-wide analysis revealed eight potential *Gli1* targets associated with myeloid cell differentiation during *H. felis* infection (Figure 4A) in addition to several inflammatory pathways (Figure S5). Of particular interest were two Schlafen genes, *Schlafen-4* (*Slfn-4*), *Schlafen-2* (*Slfn-2*) and *serum amyloid protein A3* (*SAA3*) (Figure 4A), whose gene products were previously implicated in macrophage differentiation [19], [20] and recruitment [21] respectively. RT-qPCR analysis confirmed that Slfn-4 and SAA3 were only induced in the *H. felis*-infected WT stomach (Figure 4B and Figure S6A). We performed *in silico* analysis to identify Gli binding sites and identified one site in the *Slfn-4* (Figure 4C), one in *Slfn-2* and several in the *SAA-3* promoters (El-Zaatari and Merchant JL, unpublished data). Chromatin immunoprecipitation (ChIP) demonstrated that Gli1 is present at the *Slfn-4* promoter near the *Gli1* binding site GGCCACCCA (consensus site  =  GACCACCCA), but not at a different site that lacked a *Gli1* binding sequence (Figure 4C). Since Slfn-4 is associated with aberrant myeloid cell differentiation [19], we further defined which myeloid subpopulations express Slfn-4^+^ in the stomach. Immunofluorescent staining (Figure 4D and Figure S6B) demonstrated that Slfn-4 was expressed in CD11b^+^CD11c^+^ myeloid cells. Flow-cytometric cell sorting coupled with RT-qPCR revealed that these CD11c^+^ myeloid cells produced significant amounts of IL-1β and TNF-α (Figure S6 C–E). Therefore, we concluded that Slfn-4 is a Gli1 gene target expressed in a specific IL-1β- and TNF-α-expressing subset of CD11b^+^CD11c^+^ myeloid population and could be used as an additional marker to identify these cells.

### Sonic Hedgehog Remains Epithelial in the Normal and Metaplastic Stomach

Although Shh ligand is secreted acutely from parietal cells within days of *Helicobacter* infection [12], the cellular source of Shh ligand responsible for Hh signaling has not been examined in the chronically infected stomach once parietal cell atrophy has occurred. We previously showed using a Shh-LacZ mouse that both surface pit and mucous neck cells express Shh despite parietal cell atrophy in 2 month *H. felis* infected mice [8]. Therefore to identify the source of Shh ligand in the 6-month infected mice, we immunostained WT and *Gli1* mutant mice with Shh antibody. Indeed, the Shh ligand was exclusively expressed by E-cadherin^+^ but not F4/80^+^ or α-SMA^+^ cells (Figure S7 A and B) and then was primarily produced by the expanded mucous neck cell population in inflamed stomachs of *H. felis*-infected WT mice (Figure S7 A–C). Therefore Shh ligand expression remained epithelial and was produced by the expanded mucous neck cell population in the chronically inflamed stomach despite parietal cell atrophy.

### Epithelial Activation of IL-6/pSTAT3 pathway in Gastric Metaplasia

**Figure 5:**
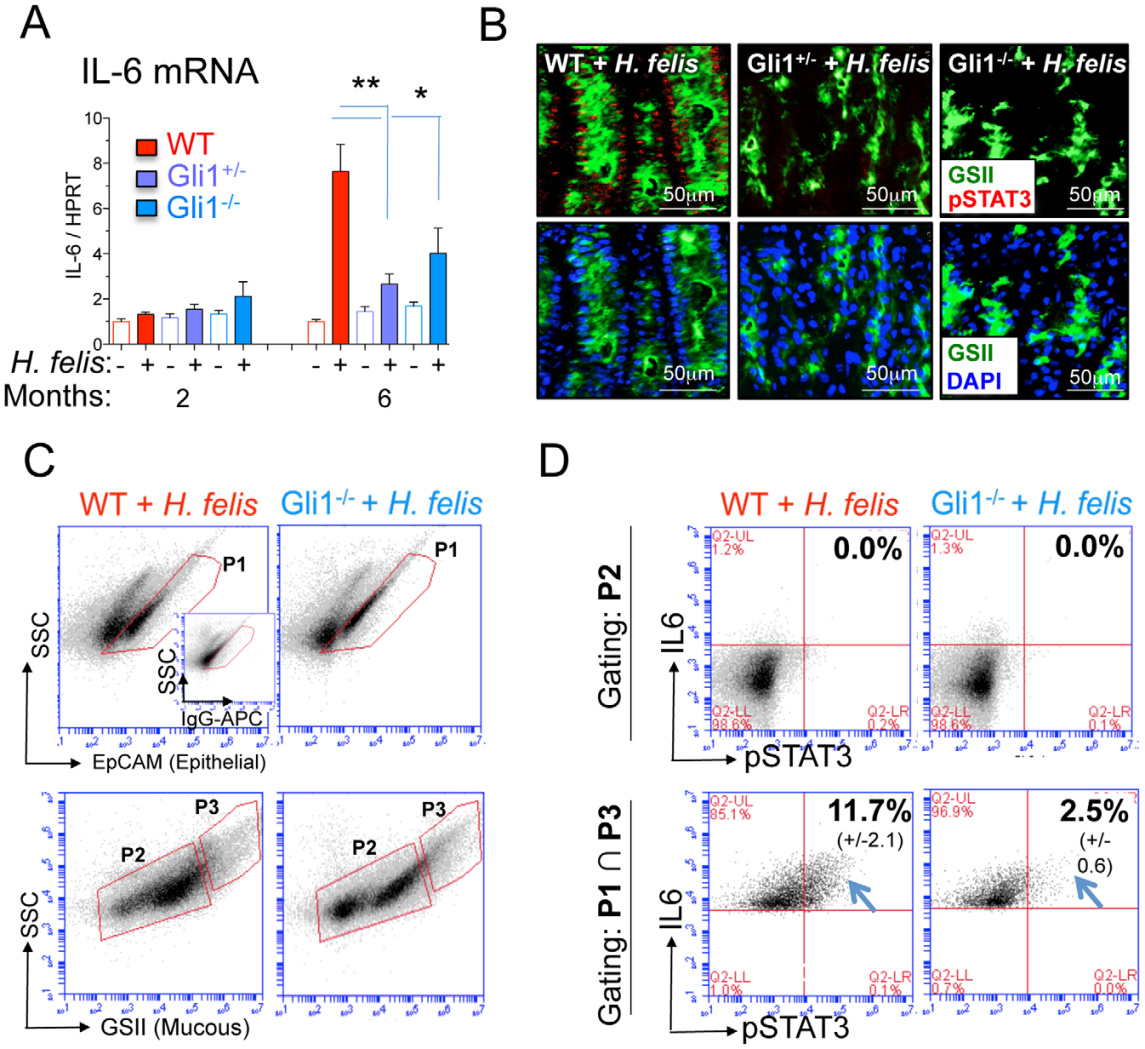
*Gli1* deletion prevents IL-6 and pSTAT3 expression in SPEM. A) RT-qPCR analysis of IL-6 mRNA. Error bars represent the mean +/− SEM. N = 5–10 mice per group. Open and closed bars denote uninfected and infected mice respectively. B) Immunofluorescent analysis of pSTAT3 (red), GSII (green) and DAPI (blue) in stomachs of 6-month infected mice. C) Flow cytometric gating for EpCAM^+^ (epithelial cells) and GSII^+^ (mucous neck cells, SPEM) in 6-month infected WT and Gli1^−/−^ mice. The inset shows the rat IgG-APC isotype control for the EpCAM-APC antibody. D) IL-6 and pSTAT3 expression in GSII^−^ (non-mucous, gate P2), and EpCAM^+^GSII^+^ (mucous neck epithelial cells, gate P1 ∩ P3) in 6-month infected WT and Gli1^−/−^ mice. Values on flow cytometric dot blots represent the mean +/− SEM from 3 mice per group. **p<0.01; *p<0.05.

Given that IL-1β induces gastric metaplasia [5], [17] and stimulates expression of the proto-oncogene IL-6 in various cell types [22], [23], [24], [25], we hypothesized that IL-1β induces expansion of the SPEM phenotype by stimulating proliferation and IL-6 expression along with phosphorylation of STAT-3 (pSTAT-3). Surprisingly, we detected IL-6 protein expression primarily in epithelial cells, particularly in metaplastic mucous cells of the *H. felis*-infected WT mice (Figure S8A) and significantly lower levels of IL-6 in the mucosa of Gli1^+/−^ and Gli1^−/−^ mice (Figure S8A). Moreover, laser capture microdissection (LCM) confirmed that most of the IL-6 expression was produced by epithelial cells after 6 months of infection (Figure S8 B and C). RT-qPCR analysis confirmed the reduction of IL-6 in infected Gli1^+/−^ and Gli1^−/−^ stomach tissue compared to WT mice (Figure 5A). The expanded population of mucous cells from the infected WT stomachs also expressed higher levels of pSTAT3 than the gastric mucosa from the Gli1^+/−^ and Gli1^−/−^ (Figure 5B). Dissociated gastric cells were analyzed by flow cytometry using an epithelial (EpCAM) and mucous neck cell marker (GSII lectin) (Figure 5C). The GSII^−^ [P2] population, which excludes mucous neck cells, was IL-6 and pSTAT3 negative (Figure 5 D, *Upper Panel*). However, a proportion of the EpCAM^+^GSII^+^ population (epithelial mucous neck cells, [P1] ∩ [P3], Figure 5C) co-expressed IL-6 and pSTAT3 (Figure 5D, *lower panel*). The proportion of epithelial mucous neck cells co-expressing IL-6 and pSTAT3 was significantly lower in infected Gli1^−/−^ mice than WT mice (Figure 5D, *lower panel*). This corroborated the immunohistochemical observation that GSII^+^ mucous neck cells expressed higher levels of pSTAT-3 in 6-month infected WT than in *Gli1*-deficient mice (Figure 5B). Consistent with the induction of the proto-oncogene pSTAT3, mucous neck cells showed higher expression of the proliferation marker Ki67 in infected WT but not in *Gli1*-deficient mice (Figure S9A). To determine whether IL-1β induced IL-6 gene expression in mucous cells, we treated the NCI-N87 gastric mucous cell line with IL-1β. IL-1β induced IL-6 gene expression in these cells and the induction was inhibited by pre-incubating with the IL-1β receptor antagonist IL-1RA (Figure S9B). Therefore, we concluded that pro-inflammatory cytokines, such as IL-1β, stimulate epithelial IL-6 expression leading to increased pSTAT3 and proliferation of the mucous cell lineage.

### Sonic Hedgehog Ligand is Not Sufficient to Induce Gastric Metaplasia

**Figure 6:**
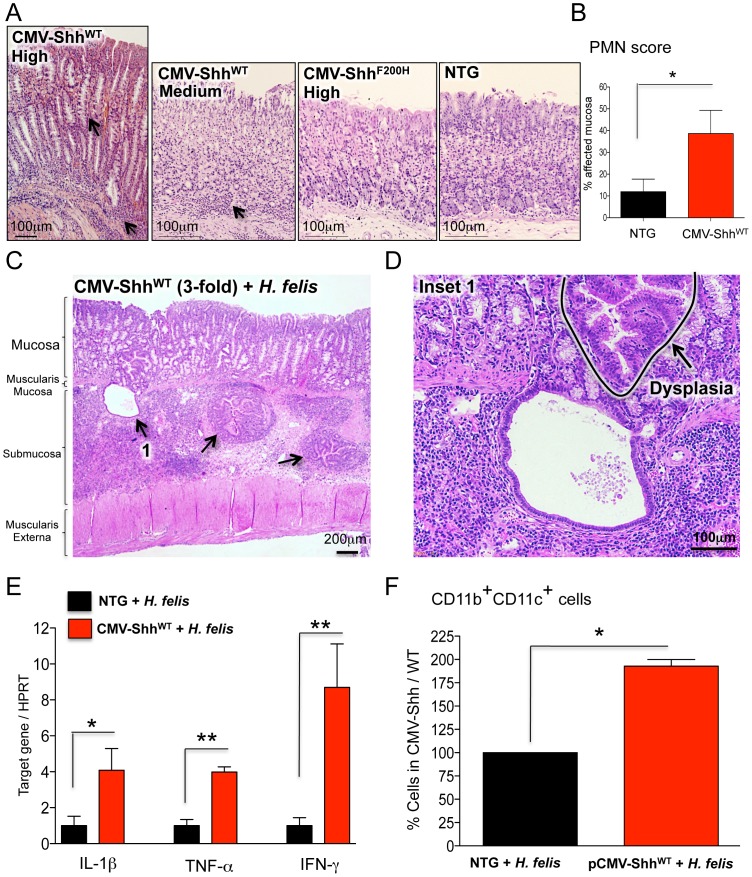
Shh overexpression exacerbates *Helicobacter* gastritis. A) H&E staining of NTG, CMV-Shh^WT^ and CMV-Shh^F200H^ mouse stomachs. Arrows indicate regions of inflammatory cell infiltration and mucous neck cell expansion, SPEM. B) Polymorphonuclear (PMN) cell scoring in NTG and CMV-Shh^WT^ mice. C) H&E staining of 6-month infected CMV-Shh^WT^ stomach. D) High power H&E staining of herniated epithelial glands in the gastric submucosa in 6-month infected CMV-Shh^WT^ stomach. A dysplastic area is indicated. E) RT-qPCR analysis of IL-1β, TNF-α and IFN-γ in 6 month-infected CMV-Shh^WT^ versus NTG stomachs. F) Bar graphs representing flow cytometric analysis of CD11b^+^CD11c^+^ cells in 6-month infected NTG and CMV-Shh^WT^ stomachs. Positive cell numbers in infected CMV-Shh^WT^ mice are expressed as a percentage of cells present in infected NTG controls (set to a 100%). Error bars represent the mean +/− SEM. N = 3 mice per group. **p<0.01; *p<0.05.

Although *Gli1* deletion demonstrated that a component of the Hh signaling pathway is necessary for inflammation and gastric metaplasia, we examined whether ectopic expression of the Shh ligand was sufficient to induce these lesions. We expressed N-terminally HA-tagged WT (Shh^WT^) and mutant (Shh^F200H^) Shh cDNA (F200H) from the CMV promoter. The Shh^F200H^ mutant cannot be cleaved to the functional 19 kDa ShhN form (Figure S10A). As previously reported [9], WT-Shh is efficiently cleaved when expressed in NIH-3T3 cells while the F200H mutant Shh only generated the uncleaved, full-length form (Figure S10B). WT-Shh induced *Gli1* and *Patched-1* (*Ptch1*) mRNA indicating active signaling, whereas the F200H form was non-functional and did not induce these two target genes (Figure S10C and S10D). Transgenic mice overexpressing CMV-Shh^WT^ and CMV-Shh^F200H^ plasmids exhibited variable transgene expression in different founder lines (Table 1). Interestingly, the CMV promoter expressed the Shh transgene exclusively in gastric chief cells (Figure S10 E and F). Overexpression of Shh^WT^ induced gastritis (3/3 medium expressing mice) at medium Shh levels and gastric metaplasia only in the highest-expressing founder (Figure 6 A and B and Table 1) characterized by the co-localization of TFF-2 and intrinsic factor (Figure S11A). By contrast, none of the CMV-Shh^F200H^ mice developed gastritis despite high expression of the transgene in some of these mice (Figure 6A and Table 1). Overexpression of WT or the F200H Shh from the parietal cell-specific H^+^/K^+^-β-ATPase promoter produced a similar phenotype to the CMV-Shh^WT^ mice marked by gastritis and infrequent metaplasia (Figure S10A, Figure S11 and Table 1). Therefore, overexpression of the Shh ligand was sufficient to induce gastric inflammation, but the presence of SPEM occurred infrequently in mice correlating with high expression of the transgene. Therefore, we concluded that Shh is sufficient to induce gastritis but not SPEM, although we cannot exclude the possibility that atrophy and metaplasia develop above a certain threshold level of Shh.

**Table 1:** Shh overexpression exacerbates *Helicobacter* gastritis.

Founders	Average Fold Expression of Shh/NTG	Histology		
		Normal	Inflammation	Metaplasia
CMV-Shh^WT^ (High Expressor)	102.0	0/1	**1/1**	**1/1**
CMV-Shh^WT^ (Medium Expressor)	6.8 (+/−1.9)	0/3	**3/3**	0/3
CMV-Shh^WT^ (Low Expressor)	0.78 (+/−0.3)	**5/5**	0/5	0/5
CMV-Shh^F200H^(High Expressor)	82.5	**1/1**	0/1	0/1
CMV-Shh^F200H^ (Medium Expressor)	19.5 (+/−9.3)	**7/7**	0/7	0/7
CMV-Shh^F200H^ (Low Expressor)	0.3	**1/1**	0/1	0/1
H^+^/K^+^-β-Shh^WT^ (Medium Expressor)	5.23 (+/−0.6)	**1/3**	**2/3**	**2/3**
H^+^/K^+^-β-Shh^WT^ (Low Expressor)	1.62 (+/−0.3)	**6/6**	0/6	0/6
H^+^/K^+^-β-Shh^F00H^ (Medium Expressor)	13	**1/1**	0/1	0/1
H^+^/K^+^-β-Shh^F200H^ (Low Expressor)	0.92 (+/−0.2)	**4/4**	0/4	0/4

* High expressor >70-fold; Medium expressor >3-fold; Low expressor <3-fold. Average transgenic expression for low-, medium- and high-expressing founders is indicated. The number of mice with normal gastric phenotype, inflammation or metaplasia is also indicated.

Since the primary phenotype of the CMV-Shh^WT^ mice was a mild gastritis, we tested whether infecting these mice with *Helicobacter* affected the severity of the inflammation and subsequent development of SPEM. CMV-Shh^WT^ mice from founder line 314 (medium transgene expression levels) and non-transgenic littermates were infected for 6 months with *H. felis*. All three CMV-Shh^WT^ mice displayed high-grade dysplasia (Figure 6 C and D), epithelial gland herniation into the submucosa (Figure 6 C and D), and elevated Th1 cytokines compared to infected non-transgenic mice (Figure 6E). Furthermore, these mice exhibited significantly higher CD11b^+^CD11c^+^ myeloid cells compared to infected non-transgenic controls (Figure 6F). Therefore, Shh overexpression exacerbated the histologic severity observed with *H. felis* infection and increased the amount of myeloid cells recruited to the inflamed stomach compared to non-transgenic mice.

## Discussion

Cancer is a sequential process initiated by epithelial cell aberrations that can be triggered and sustained by immune and stromal cell types within the tumor microenvironment [26], [27]. In the stomach, infection by *Helicobacter* is correlated with the development of gastric cancer in the setting of chronic atrophic gastritis [28]. Since Hh signaling has been linked to a variety of cancers, some of which develop in the setting of inflammation [29], [30], [31], we examined the role of Gli1, a known component of the Hh signaling pathway, in the transition from chronic inflammation to mucous neck cell metaplasia or SPEM. Gli1 is expressed in both intestinal myeloid and myofibroblasts [11], consistent with prior studies showing Gli1 in hematopoietic stem cells [32], [33]. We can only speculate as to why Gli1 is lost only in α-SMA^+^ cells of the muscularis mucosa in response to *H. felis*, but not in the intraglandular mesenchyme. Nevertheless similar changes occurred in the H^+^/K^+^-ATPase-IFN-γ mice [34], and might pertain to the heterogeneity of the α-SMA^+^ cells responding to the inflammatory cytokines [5].

In the current study, we examined the role of Gli1 during chronic *Helicobacter* infection and found that Gli1 was required for SPEM to develop. In addition, the inflammation correlated with infiltration of a specific subpopulation of myeloid cells. SPEM arose in response to *Helicobacter*-induced expansion of the mucous neck cell compartment and acquired proliferative markers including Ki67, IL-6 and pSTAT3. Examining Gli1-dependent myeloid cell subpopulations that correlated with the metaplastic phenotype, we found that these populations were CD45^+^MHCII^+^CD11b^+^CD11c^+^. Interestingly, the myeloid subpopulation that expressed CD11c only appeared in the stomach and not in the bone marrow, suggesting that the acquisition of the CD11c surface marker occurs within the inflamed gastric environment. However, additional phenotypic and functional characterization of this CD11b^+^CD11c^+^ population requires their direct isolation. Since using Gli1 antibodies to sort for these myeloid subsets is not currently feasible, we used genome-wide analysis to identify Gli1 gene targets in myeloid cells, e.g., Slfn-4, which might be used to isolate these cells for further study. We demonstrated here that Slfn-4 contains a Gli1 binding site in its promoter. Moreover, Slfn4 expression has been linked to aberrant myelopoiesis [19], making it a relevant regulatory gene contributing to generation of the various Gli1-dependent myeloid populations during *Helicobacter* infection. Therefore, the current phenotype of the myeloid subset correlating with SPEM development is a CD45^+^MHCII^+^CD11b^+^CD11c^+^Slfn4^+^ cell that expresses IL-1β and TNFα. Although this phenotype is consistent with an “inflammatory monocyte” activated in the periphery by tissue damage and inflammation [18], [35], the actual function of these cells awaits their isolation and further characterization including chemokine profile and possible suppressor activity.

Although Shh ligand induces Gli1 expression, we previously reported that Shh is lost from parietal cells in the presence of inflammatory cytokines [8]. Indeed reduced Shh expression has been reported in *H. pylori*-infected human subjects [36]. Nevertheless we show here that metaplastic mucous cells express Shh ligand in the absence of parietal cells. In a study of myeloid cell progenitors using Gli1LacZ mice, Merchant et al. demonstrate loss of hematopoietic stem cell proliferation in Gli1 null mice and suggest that non-Hh ligand mechanisms regulate bone marrow myeloid cell populations [33]. Likewise, we also found no evidence for ligand-mediated induction of Gli1 in RAW 264.7 cells, a mouse myeloid cell line (El-Zaatari and Merchant JL, unpublished data). Indeed, ectopic Shh expression was generally not sufficient to induce metaplasia in the absence of the *Helicobacter* – mediated inflammation. This result was consistent with Gli1 regulation of myeloid subpopulations being required but not ligand-dependent. Thus while parietal cell secretion of Shh into the circulation is important for the initial recruitment of inflammatory cells to the stomach [12], the ligand does not appear to mediate further myeloid cell differentiation ostensibly required for the pre-neoplastic changes.

We also characterized the mechanism by which IL-1β induces mucous cell expansion and metaplasia. A recent study shows that IL-6 mediates IL-1β induction of Barrett's metaplasia of the esophagus [25]. IL-6 binds the gp130 receptor [37] and induces the proto-oncogene pSTAT3 [38], which stimulates proliferation. It is well documented that activation of the gp130 cytokine receptor is sufficient to mediate gastric cancer [39]. These studies also demonstrated that IL-11, rather than IL-6, mediates the effects of gp130 activation on gastric tumorigenesis [39]. However, transfer of bone marrow cells exhibiting overactive gp130 is not sufficient to induce gastric cancer indicating that gp130-induced gastric carcinogenesis is epithelial cell-mediated [39], and might represent a distinct mechanism from *Helicobacter*-induced carcinogenesis. Administration of IL-11 induces foveolar gastric hyperplasia but does not induce SPEM [40]. Therefore in contrast to gp130-mediated antral tumors driven by the IL-6 family member IL-11, we show here that *Helicobacter* infection drives IL-6 expression and SPEM. Surprisingly, the expanded mucous epithelial cells of the stomach corpus were a major source of IL-6. Correlating with increased IL-6 production by mucous cells, these cells expressed elevated levels of pSTAT3 and were highly proliferative. Indeed, IL-1β induced IL-6 expression in gastric mucous cells suggesting that myeloid cell-secreted cytokines target epithelial cells. This data demonstrated that stromal signals from myeloid cells activate the proliferation of gastric epithelial cells leading to SPEM.

**Figure 7:**
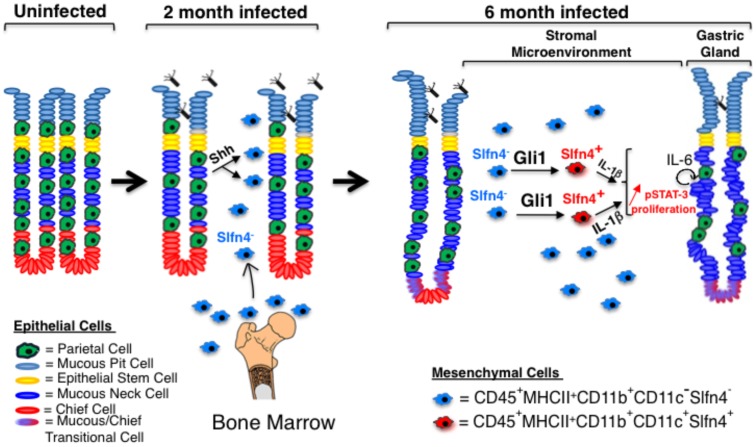
Proposed role of Hedgehog-dependent myeloid cells in *Helicobacter*-induced gastric metaplasia. Upon *Helicobacter* infection, CD11b^+^CD11c^−^Slfn-4^−^ myeloid cells migrate from the bone marrow to the stomach in response to Shh ligand secretion from parietal cells. Prolonged exposure of these cells to the inflamed gastric environment induced by *H. felis* (∼6 months) induces a phenotypic shift into CD11b^+^CD11c^+^ myeloid cells, which express the marker Slfn-4. Gli1 expression in myeloid cells is necessary for this shift. Myeloid cell-derived IL-1β triggers the IL-6/pSTAT-3 pathway in epithelial mucous cells leading to mucous neck cell proliferation at the expense of parietal and chief cells (gland atrophy) and metaplasia (SPEM).

As suggested by Merchant et al., Gli1 is not required for myeloid cell development or maintenance but rather is required when stress-induced hematopoiesis increases the common progenitor pool of cells that differentiate into specific myeloid subsets [33]. We conclude that in the stomach, this pathway has two phases (Figure 7). First, bacterial infection induces the production and release of Shh ligand to stimulate the recruitment of Gli1^+^ myeloid cells to the stomach [12]. However during chronic infection, these same Gli1^+^ cells acquire new markers through Hh ligand-independent mechanisms that promote metaplasic, proliferative changes in the gastric epithelium, setting the stage for neoplastic transformation.

## Supporting Information

Figure S1 **Infected wild-type mice develop gastric SPEM.** *Left Panel*, Confocal imaging of trefoil factor 2 (TFF-2, green) and DAPI (blue) in the stomach of 6-month *H. felis*-infected wild-type mice. *Insets* (high power), co-immunofluorescent imaging of TFF-2 (green) and intrinsic factor (red), in 6-month *H. felis*-infected wild-type mice with and without DAPI (blue, *middle and left panels* respectively). Numbers on insets indicate magnified regions.
(TIF)

Figure S2 **Infiltrating myeloid cells express Gli1.** A) Immunofluorescent analysis of β-gal (red), F4/80 (green), and DAPI (blue) in H. felis-infected Gli1^+/LacZ^ mice. B) Immunofluorescent detection of β-gal (red), CD11b (green), and DAPI (blue) in H. felis-infected Gli1^+/LacZ^ mice. C) Immunofluorescent detection of β-gal (red), CD11c (green), and DAPI (blue) in *H. felis*-infected Gli1^+/LacZ^ mice. Analysis was performed in 6-month infected mice. Numbers on insets indicate magnified regions.
(TIF)

Figure S3 **B cells, T cells, and neutrophils do not express Gli1.** A) Immunofluorescent analysis of β-gal (red), CD19 (green), and DAPI (blue). B) Immunofluorescence of β-gal (red), CD3 (green) and DAPI (blue). C) Immunofluorescence of β-gal (red), MPO (green), and DAPI (blue). D) Concentration-matched hamster, rat and rabbit isotype controls. E) Immunofluorescent analysis of β-gal expression in Gli1^+/LacZ^ versus WT mice. Numbers on insets indicate magnified regions.
(TIF)

Figure S4 ***Gli1*** **deletion prevents CD11c^+^ cell expansion in 6-month** ***H. felis*** **-infected stomachs.** Confocal imaging of CD11b (green, *top panel*), CD11c (green, *lower panel*) and DAPI (blue) in 6-month *H. felis*-infected WT, Gli1^+/−^ and Gli1^−/−^ stomachs.
(TIF)

Figure S5 ***Gli1*** **deletion prevents the induction of pro-inflammatory genes in** ***H. felis*** **-infected stomachs at after 6 months of infection.** Microarray heat map of *H. felis*-induced and Gli1-dependent gene groups in the stomach. Each column represents pooled stomach RNA samples from two mice.
(TIF)

Figure S6 **Gli1 is required for the appearance of a Slfn-4^+^ population that produces IL-1β and TNF-α.** A) RT-qPCR analysis of *SAA3* mRNA from sham- or 6 month *H. felis*-infected WT, Gli1^+/−^, or Gli1^−/−^ stomachs. B) Immunofluorescent analysis of CD11c (green) and Slfn-4 (red) in 6-month infected WT mice. C) Flow cytometric analysis of F4/80 and CD11c in 6-month infected WT mouse stomachs. D) Flow cytometric analysis of F4/80 and CD11c subtypes in CD45^+^Slfn4^+^ myeloid cells from 6-month infected WT mouse stomachs. F) Semi-quantitative RT-PCR analysis of flow sorted F4/80^−^CD11c^−^ (P9) versus F4/80^+^CD11c^+^ (P3) cells. Numbers on insets indicate magnified regions. Arrows indicate co-localization of Slfn4, CD11b and CD11c markers.
(TIF)

Figure S7 **Epithelial cells express Shh ligand.** A) Triple immunofluorescent staining of Shh (red), E-cadherin (epithelial, green) and F4/80 (mesenchymal, grey) in sham- and *H. felis*-infected WT, Gli1^+/−^ and Gli1^−/−^ mice. B) Double immunofluorescent staining of Shh (red) and α-SMA (green). C) Double staining of Shh (red) and GSII (green). White arrows indicate the expanded mucous neck cell lineage in *H. felis*-infected WT mice at 6 months.
(TIF)

Figure S8 ***Gli1*** **deletion prevents mucous neck cell compartment expansion and IL-6 production.** A) Immunofluorescent analysis of GSII (green) and IL-6 (red) in 6-month infected mice. B) Laser Capture Microdissection of toluidine stained section. Shown are the micrographs showing the epithelial (*top panel*) versus stromal regions (*lower panel*) from a 6-month infected WT stomach with SPEM. C) Semi-quantitative RT-PCR analysis of IL-6 in epithelial versus stromal laser microdissectates.
(TIF)

Figure S9 **Gli1 and IL-1β mediate mucous cell proliferation and IL-6 production by mucous cells respectively.** A) Immunofluorescent staining for Ki67 (red) and GSII (green)-positive cells in 6-month infected stomachs. (B) RT-qPCR analysis of IL-6 in NCI-N87 cells treated with or without IL-6, IL-1β, and IL-1RA. Error bars represent the mean +/− SEM. *p<0.05.
(TIF)

Figure S10 **Transgene expression of Shh ligand in mice.** A) Diagrammatic representation of pCMV-Shh^WT^, pCMV-Shh^F200H^, H^+^/K^+^-ATPase-β-Shh^WT^, and H^+^/K^+^-ATPase-β-Shh^F200H^ constructs. The HA tag is indicated. B) Western blotting analysis of NIH-3T3 conditioned media following transfection with pCMV-Shh^WT^ and pCMV-Shh^F200H^. C–D) RT-qPCR of Gli1 and Ptch1 mRNA expression in NIH-3T3 cells transfected with pCMV-Shh^WT^ or pCMV-Shh^F200H^ plasmids. (E) *Upper Panel*: Immunofluorescent staining of GSII (green) and HA-tag (red) in NTG, pCMV-Shh^WT-HA^, and pCMV-Shh^F200H-HA^ mouse stomachs. *Middle Panel*: Shh (green) and H^+^/K^+^-ATPase (red). *Lower Panel*: intrinsic factor (IF; green) and HA-tag (red). (F) Immunofluorescent staining of Shh (green) and HA-tag (red) in pCMV-Shh^WT^ and pCMV-Shh^F200H^ mouse stomachs. A short 15 min incubation with Shh antibody was used to detect transgenes overexpressing Shh^WT^ and Shh^F200H^ without cross-reacting with endogenous gastric Shh. Error bars represent the mean +/− SEM. *p<0.05.
(TIF)

Figure S11 **SPEM in pCMV-Shh^WT^ and H^+^/K^+^-ATPase-β-Shh^WT^ mice.** A) *Left panel*, Immunofluorescent staining of TFF-2 (green), intrinsic factor (IF, red), and DAPI (blue) in the highest expressing pCMV-Shh^WT^ mouse stomach (102-fold expression of Shh, please refer to Table 1); *right panel*, high power magnification of the co-localization of IF (red) and TFF-2 (green). B) H&E staining of mouse stomach in two H^+^/K^+^-ATPase-β-Shh^WT^ founder mice expressing medium levels of the transgene (please refer to Table 1). C) *Left panel*, immunofluorescent staining of TFF-2 (green), intrinsic factor (IF, red), and DAPI (blue) in an H^+^/K^+^-ATPase-β-Shh^WT^ founder mouse expressing medium levels of the transgene (please refer to Table 1); *right panel*, high power magnification of the co-localization of IF (red) and TFF-2 (green). Arrows indicate regions of inflammatory cell infiltration and mucous neck cell expansion.
(TIF)
