# Supplementary figures and images for: Gli1 Deletion Prevents Helicobacter-Induced Gastric Metaplasia and Expansion of Myeloid Cell Subsets
Source: PLoS One. 2013 Mar 8;8(3):e58935. doi: 10.1371/journal.pone.0058935 (PMC3592845; doi:10.1371/journal.pone.0058935)

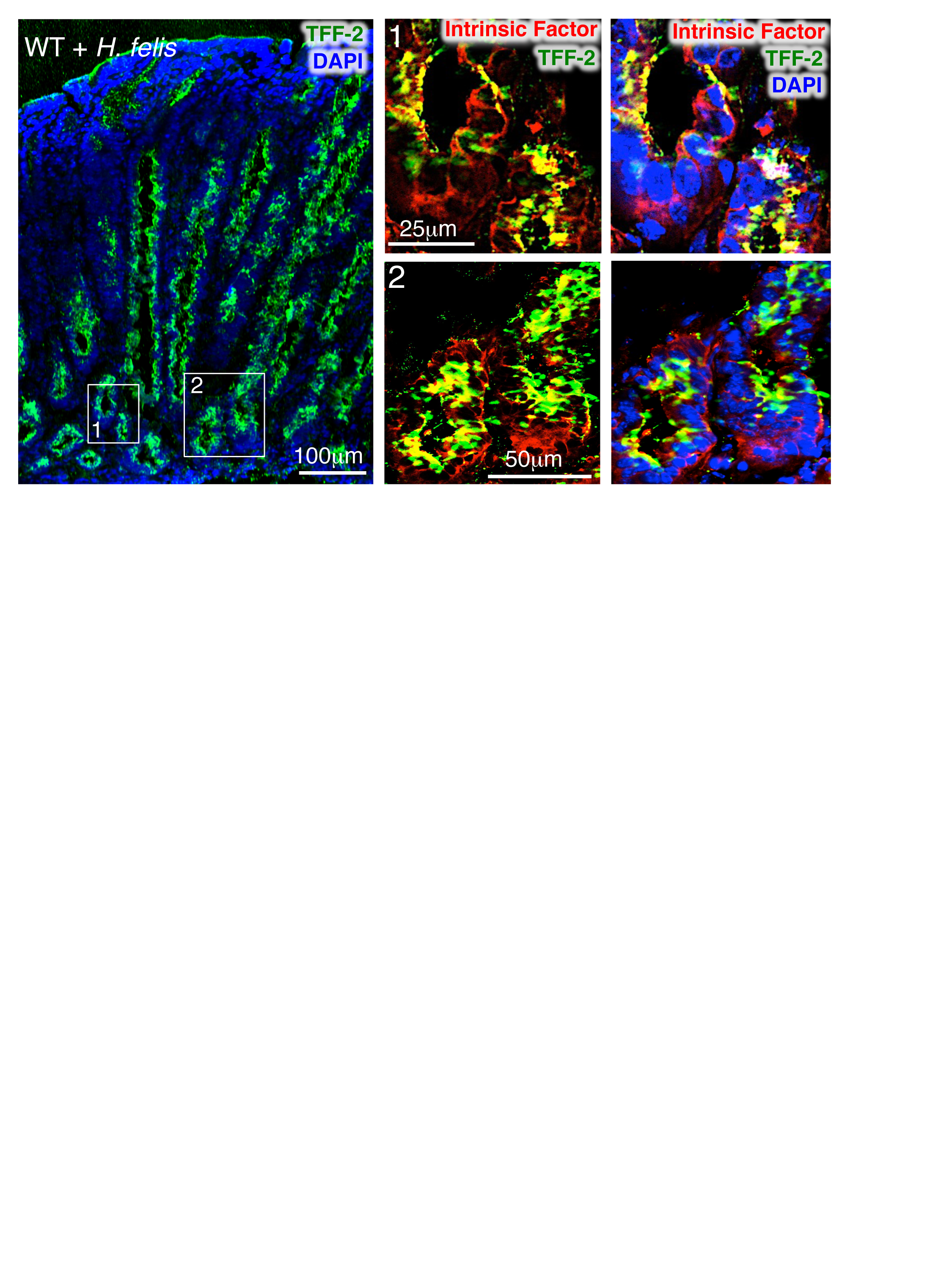

Supplement: Figure S1 — Infected wild-type mice develop gastric SPEM. Left Panel, Confocal imaging of trefoil factor 2 (TFF-2, green) and DAPI (blue) in the stomach of 6-month H. felis-infected wild-type mice. Insets (high power), co-immunofluorescent imaging of TFF-2 (green) and intrinsic factor (red), in 6-month H. felis-infected wild-type mice with and without DAPI (blue, middle and left panels respectively). Numbers on insets indicate magnified regions. (TIF) [file pone.0058935.s001.tif]

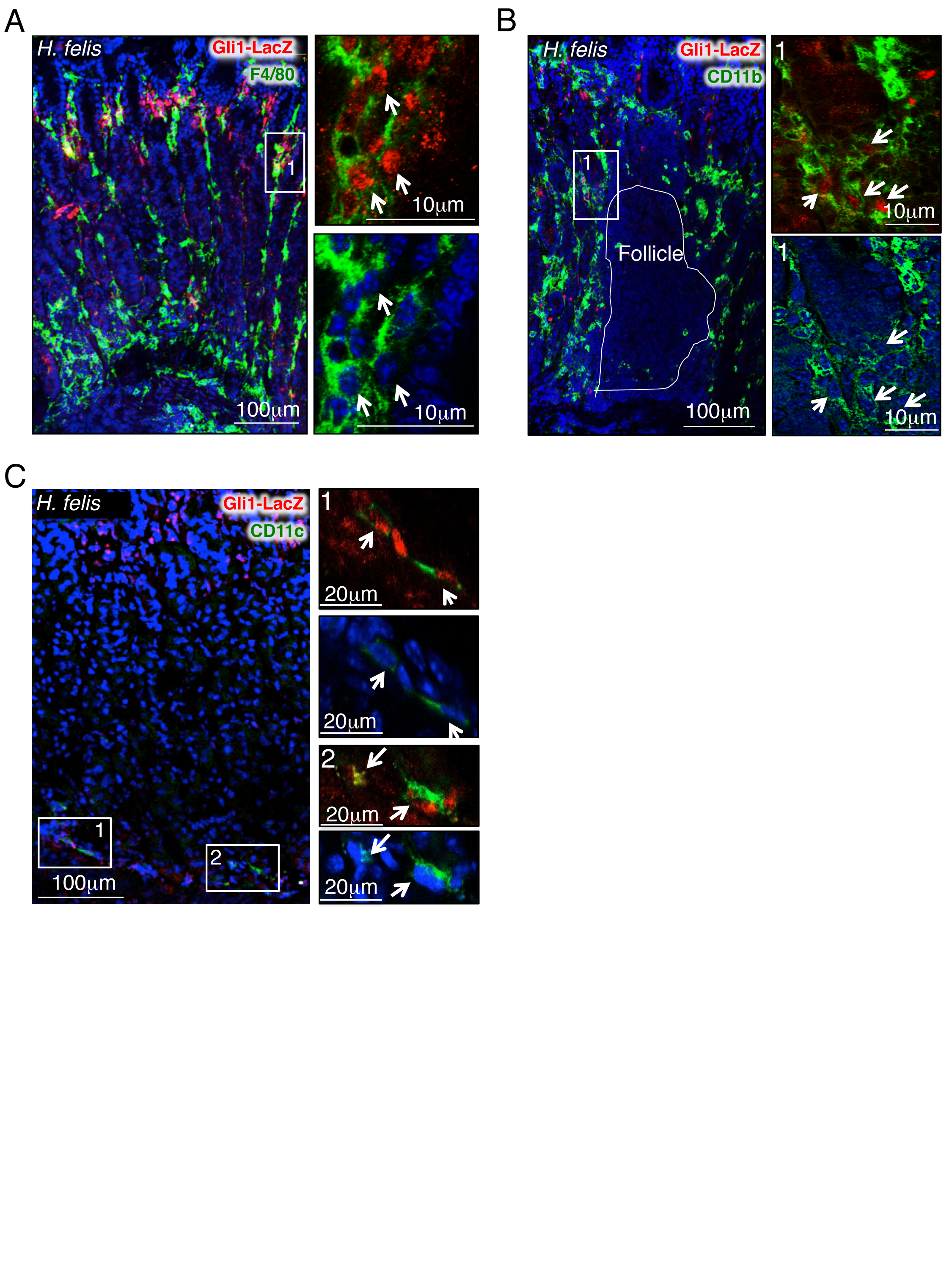

Supplement: Figure S2 — Infiltrating myeloid cells express Gli1. A) Immunofluorescent analysis of β-gal (red), F4/80 (green), and DAPI (blue) in H. felis-infected Gli1+/LacZ mice. B) Immunofluorescent detection of β-gal (red), CD11b (green), and DAPI (blue) in H. felis-infected Gli1+/LacZ mice. C) Immunofluorescent detection of β-gal (red), CD11c (green), and DAPI (blue) in H. felis-infected Gli1+/LacZ mice. Analysis was performed in 6-month infected mice. Numbers on insets indicate magnified regions. (TIF) [file pone.0058935.s002.tif]

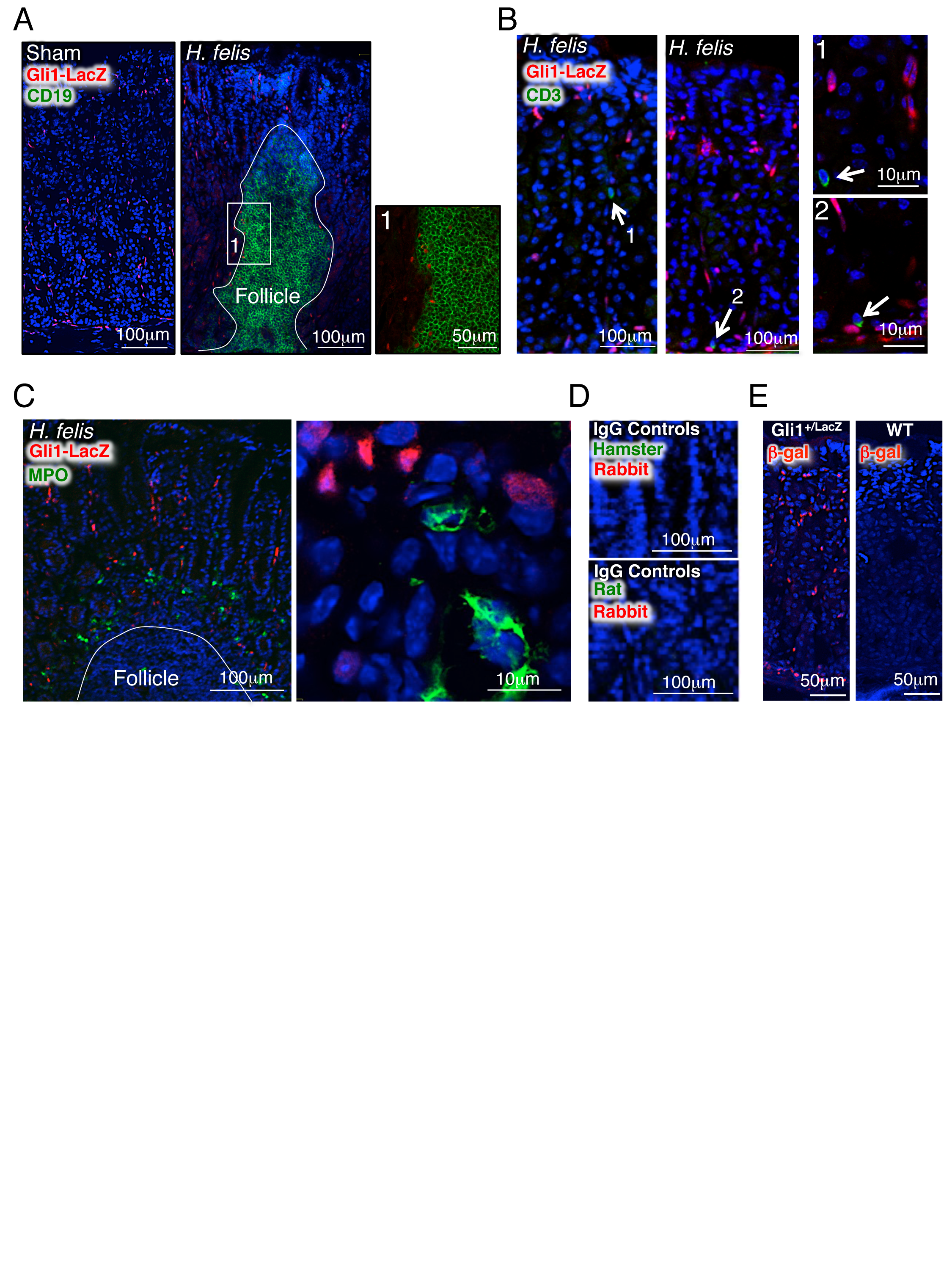

Supplement: Figure S3 — B cells, T cells, and neutrophils do not express Gli1. A) Immunofluorescent analysis of β-gal (red), CD19 (green), and DAPI (blue). B) Immunofluorescence of β-gal (red), CD3 (green) and DAPI (blue). C) Immunofluorescence of β-gal (red), MPO (green), and DAPI (blue). D) Concentration-matched hamster, rat and rabbit isotype controls. E) Immunofluorescent analysis of β-gal expression in Gli1+/LacZ versus WT mice. Numbers on insets indicate magnified regions. (TIF) [file pone.0058935.s003.tif]

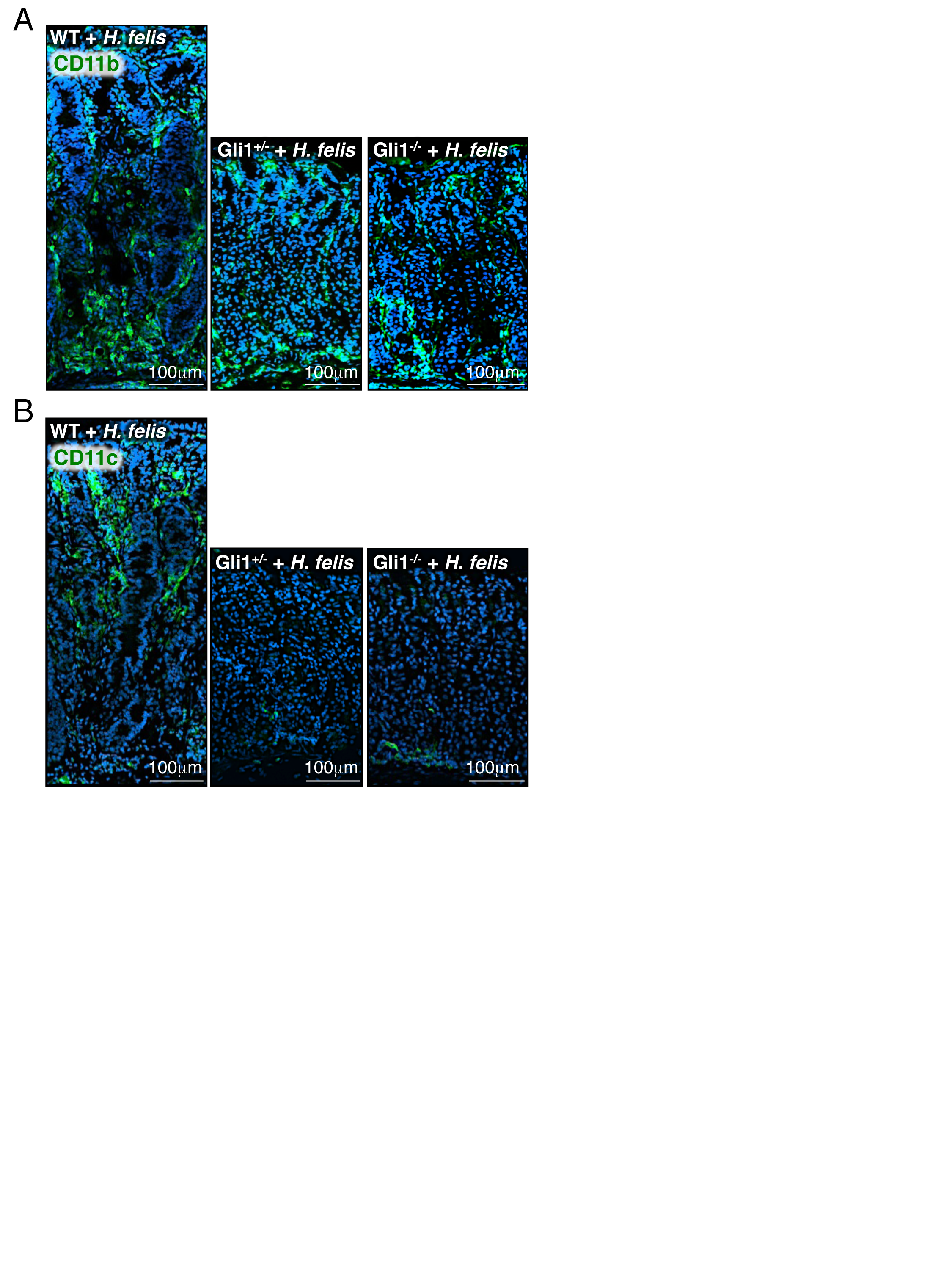

Supplement: Figure S4 — Gli1 deletion prevents CD11c+ cell expansion in 6-month H. felis -infected stomachs. Confocal imaging of CD11b (green, top panel), CD11c (green, lower panel) and DAPI (blue) in 6-month H. felis-infected WT, Gli1+/− and Gli1−/− stomachs. (TIF) [file pone.0058935.s004.tif]

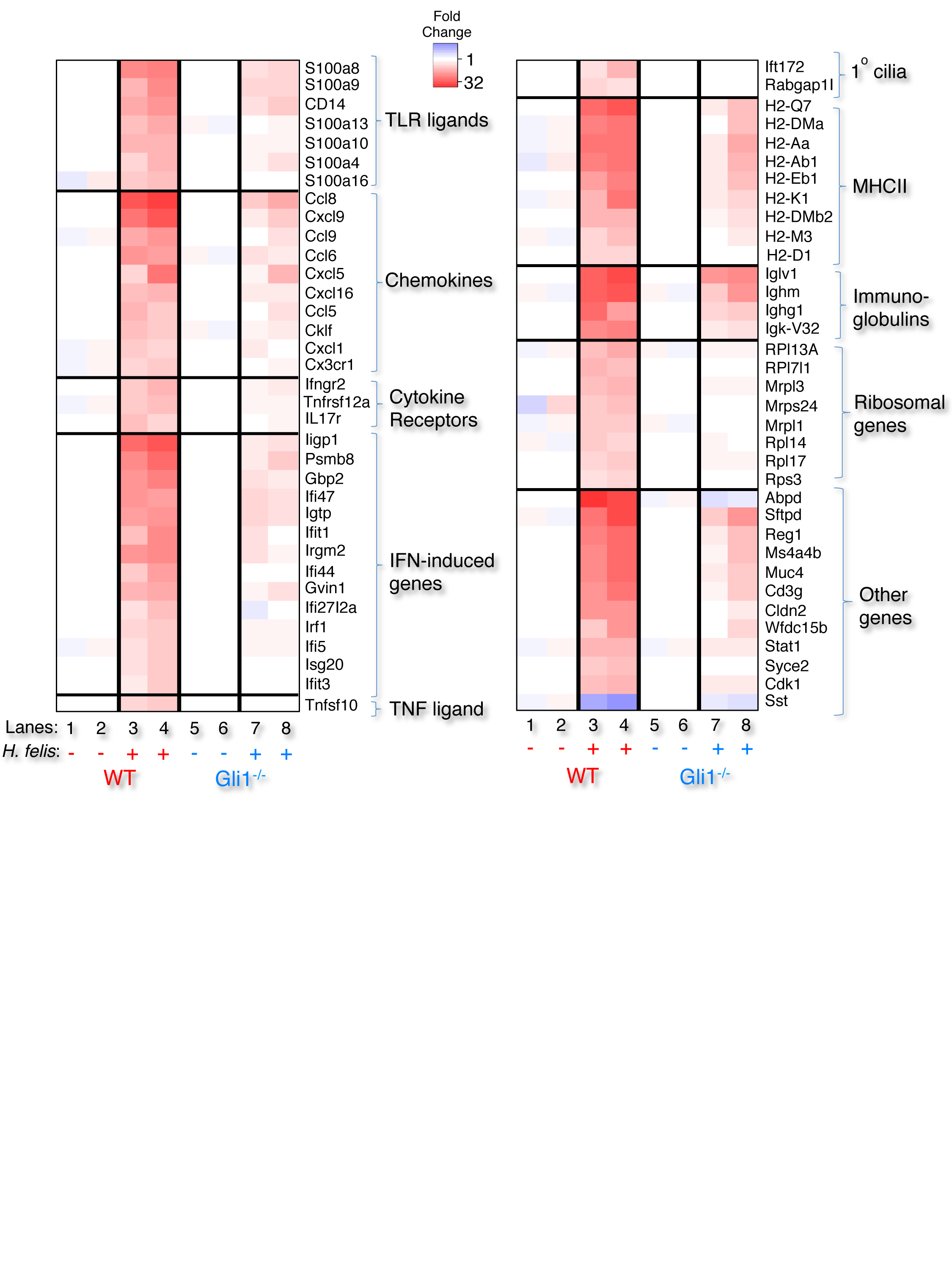

Supplement: Figure S5 — Gli1 deletion prevents the induction of pro-inflammatory genes in H. felis -infected stomachs at after 6 months of infection. Microarray heat map of H. felis-induced and Gli1-dependent gene groups in the stomach. Each column represents pooled stomach RNA samples from two mice. (TIF) [file pone.0058935.s005.tif]

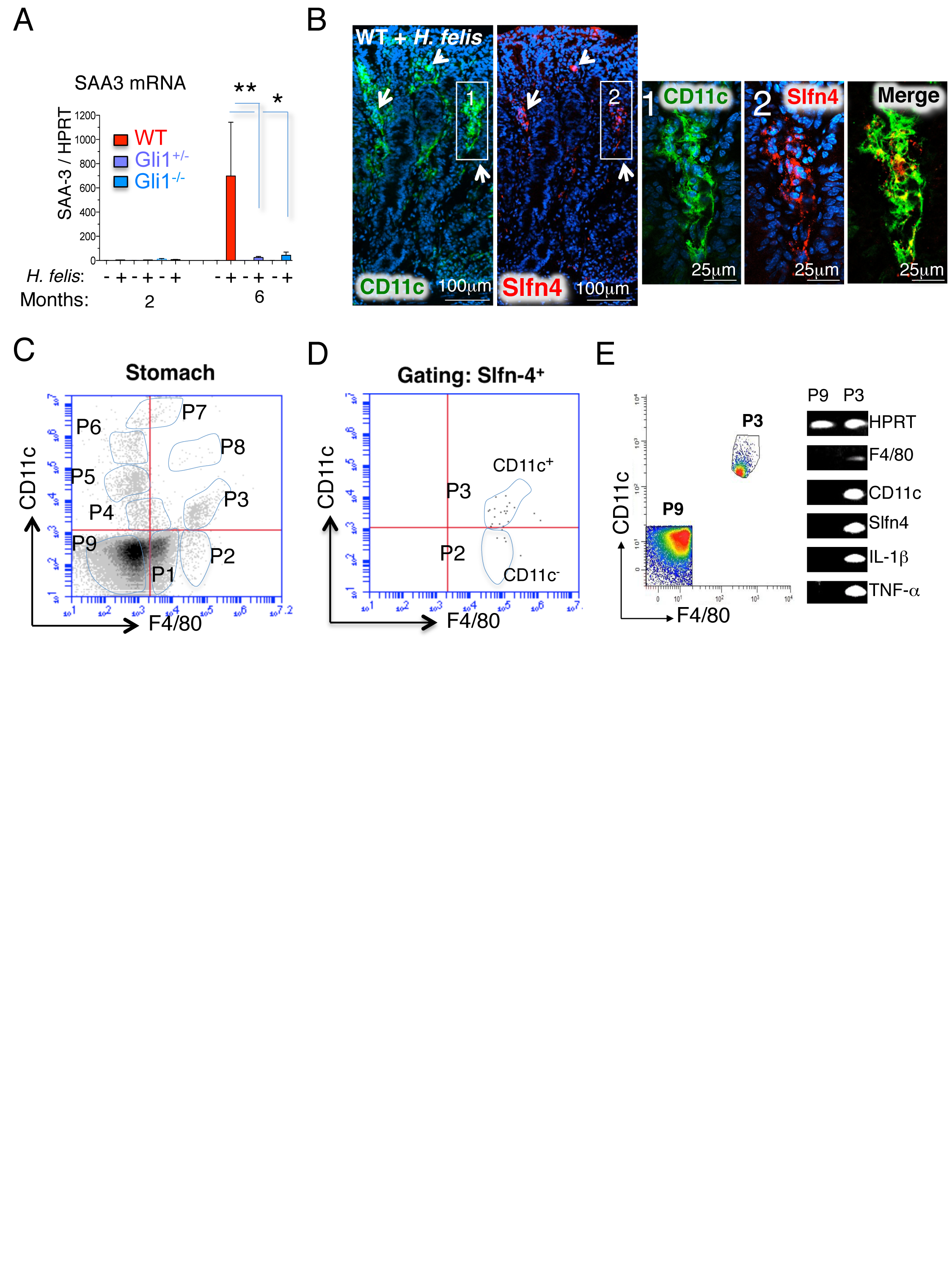

Supplement: Figure S6 — Gli1 is required for the appearance of a Slfn-4+ population that produces IL-1β and TNF-α. A) RT-qPCR analysis of SAA3 mRNA from sham- or 6 month H. felis-infected WT, Gli1+/−, or Gli1−/− stomachs. B) Immunofluorescent analysis of CD11c (green) and Slfn-4 (red) in 6-month infected WT mice. C) Flow cytometric analysis of F4/80 and CD11c in 6-month infected WT mouse stomachs. D) Flow cytometric analysis of F4/80 and CD11c subtypes in CD45+Slfn4+ myeloid cells from 6-month infected WT mouse stomachs. F) Semi-quantitative RT-PCR analysis of flow sorted F4/80−CD11c− (P9) versus F4/80+CD11c+ (P3) cells. Numbers on insets indicate magnified regions. Arrows indicate co-localization of Slfn4, CD11b and CD11c markers. (TIF) [file pone.0058935.s006.tif]

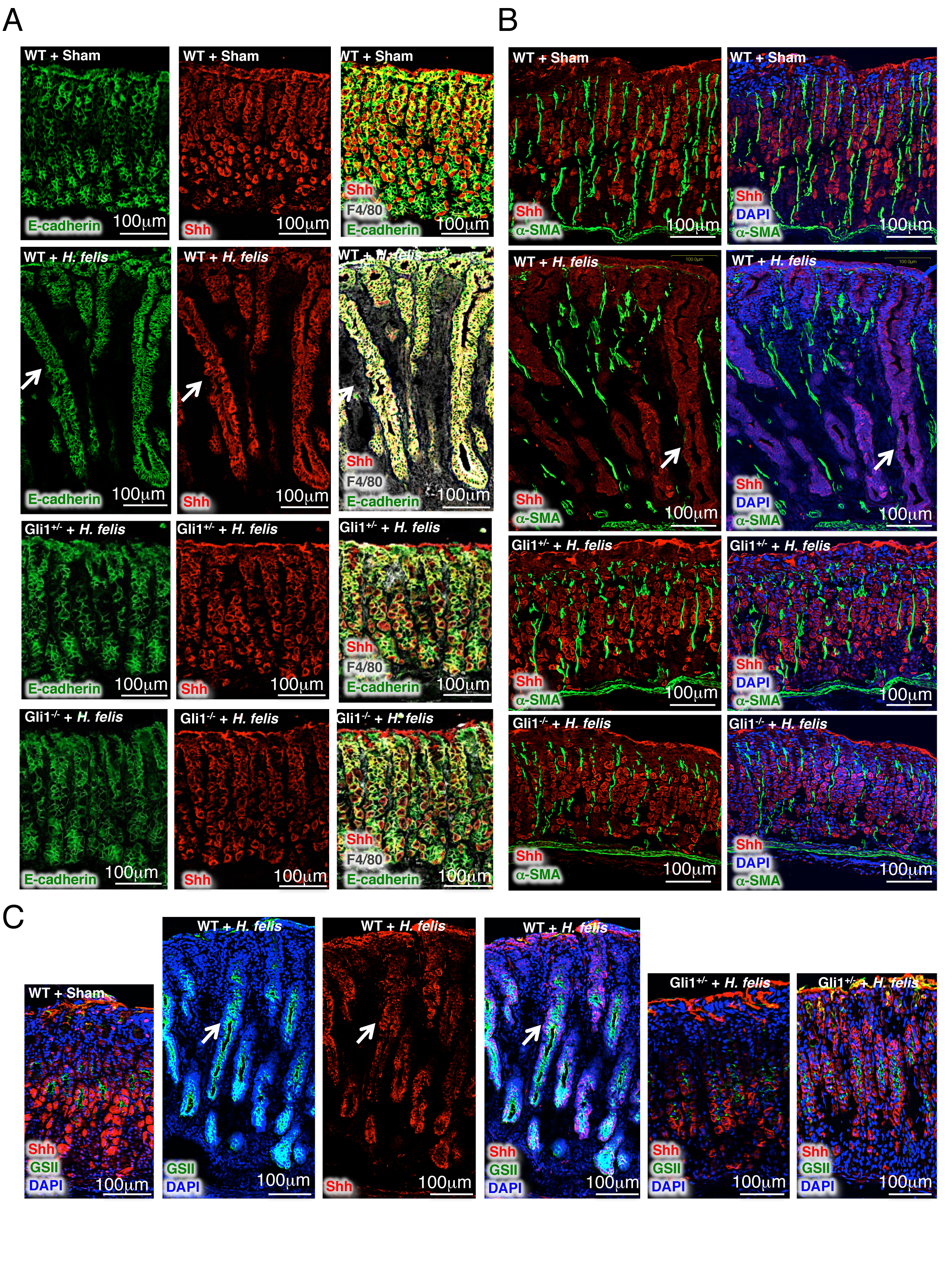

Supplement: Figure S7 — Epithelial cells express Shh ligand. A) Triple immunofluorescent staining of Shh (red), E-cadherin (epithelial, green) and F4/80 (mesenchymal, grey) in sham- and H. felis-infected WT, Gli1+/− and Gli1−/− mice. B) Double immunofluorescent staining of Shh (red) and α-SMA (green). C) Double staining of Shh (red) and GSII (green). White arrows indicate the expanded mucous neck cell lineage in H. felis-infected WT mice at 6 months. (TIF) [file pone.0058935.s007.tif]

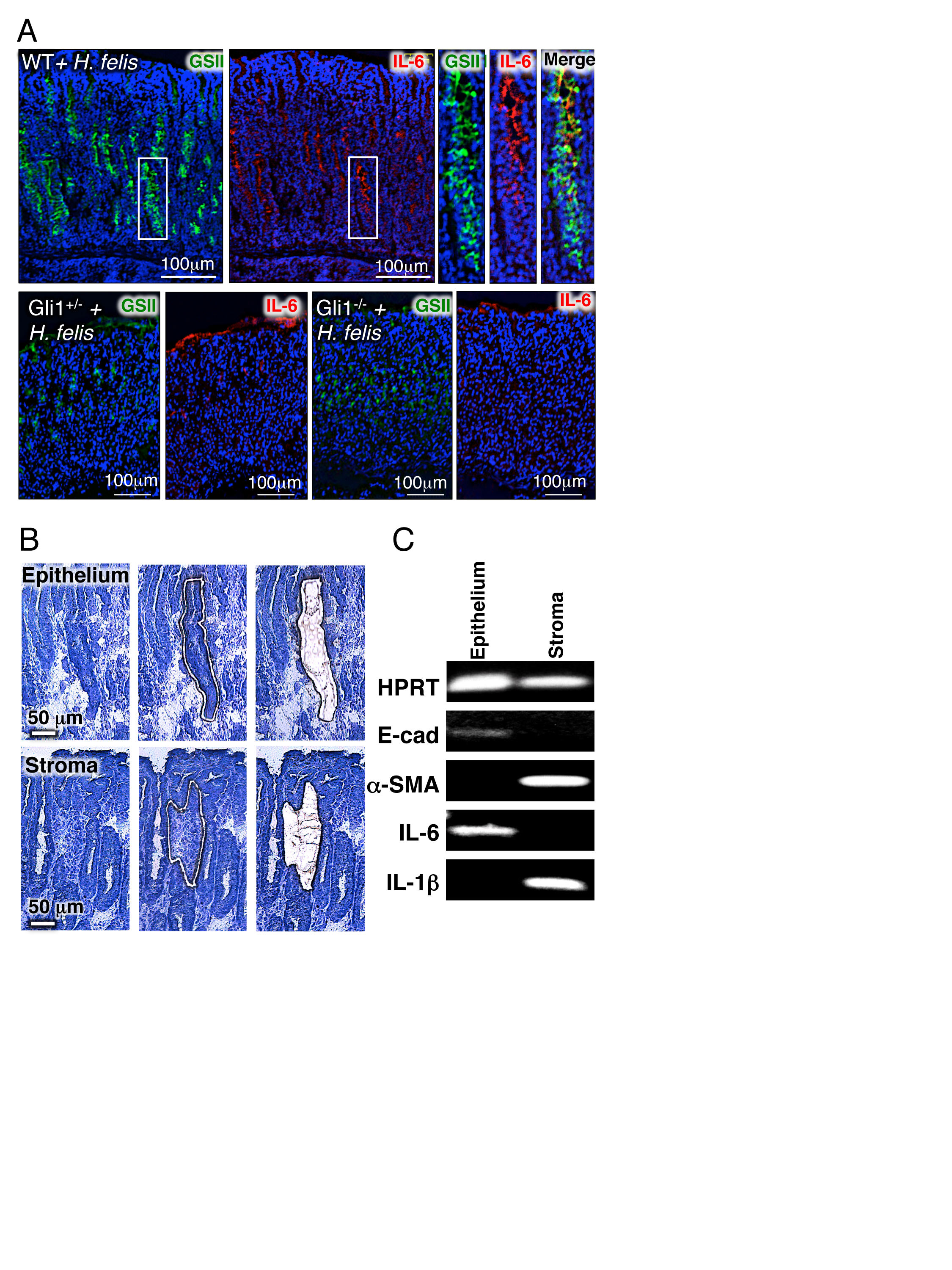

Supplement: Figure S8 — Gli1 deletion prevents mucous neck cell compartment expansion and IL-6 production. A) Immunofluorescent analysis of GSII (green) and IL-6 (red) in 6-month infected mice. B) Laser Capture Microdissection of toluidine stained section. Shown are the micrographs showing the epithelial (top panel) versus stromal regions (lower panel) from a 6-month infected WT stomach with SPEM. C) Semi-quantitative RT-PCR analysis of IL-6 in epithelial versus stromal laser microdissectates. (TIF) [file pone.0058935.s008.tif]

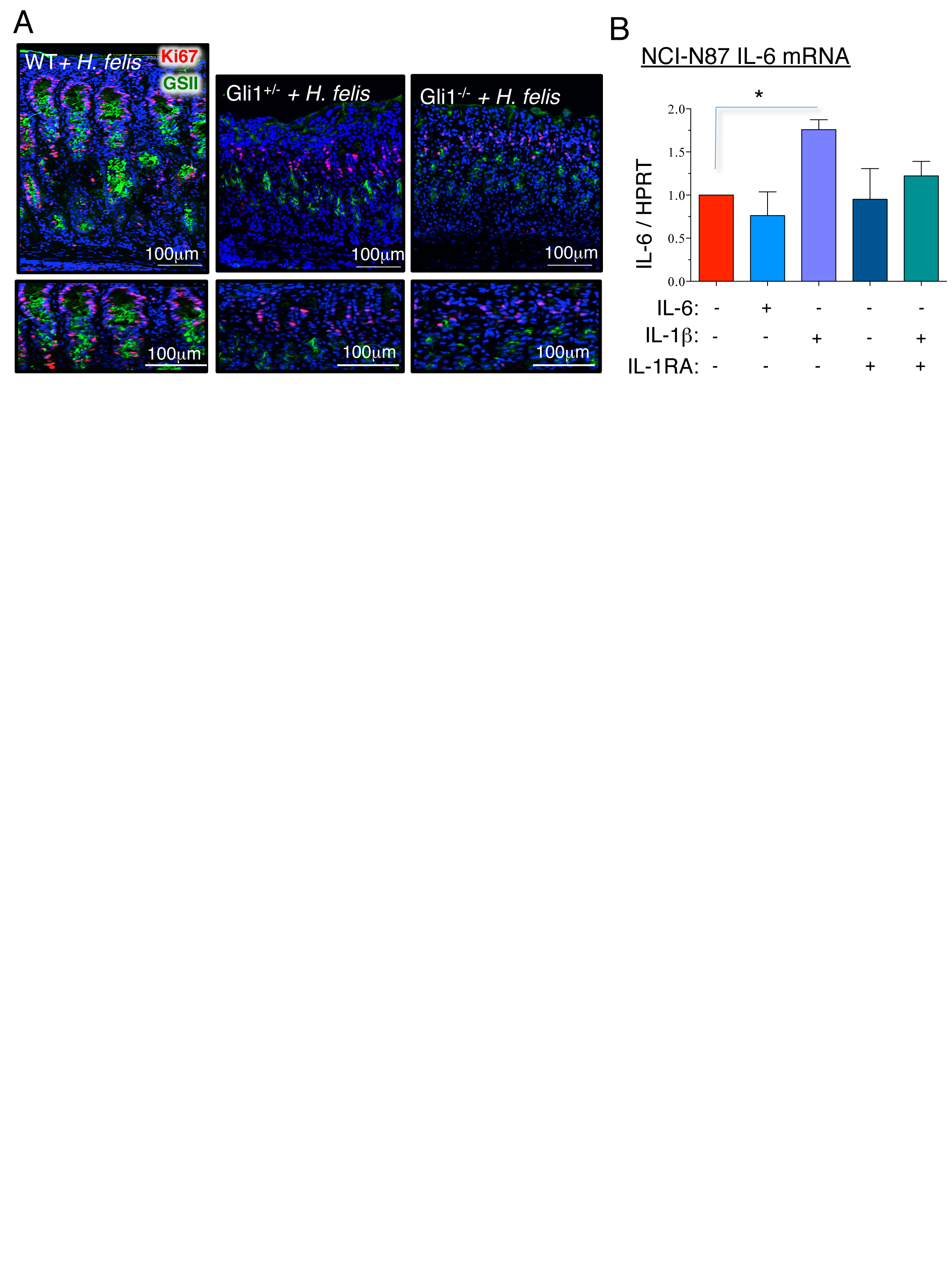

Supplement: Figure S9 — Gli1 and IL-1β mediate mucous cell proliferation and IL-6 production by mucous cells respectively. A) Immunofluorescent staining for Ki67 (red) and GSII (green)-positive cells in 6-month infected stomachs. (B) RT-qPCR analysis of IL-6 in NCI-N87 cells treated with or without IL-6, IL-1β, and IL-1RA. Error bars represent the mean +/− SEM. *p<0.05. (TIF) [file pone.0058935.s009.tif]

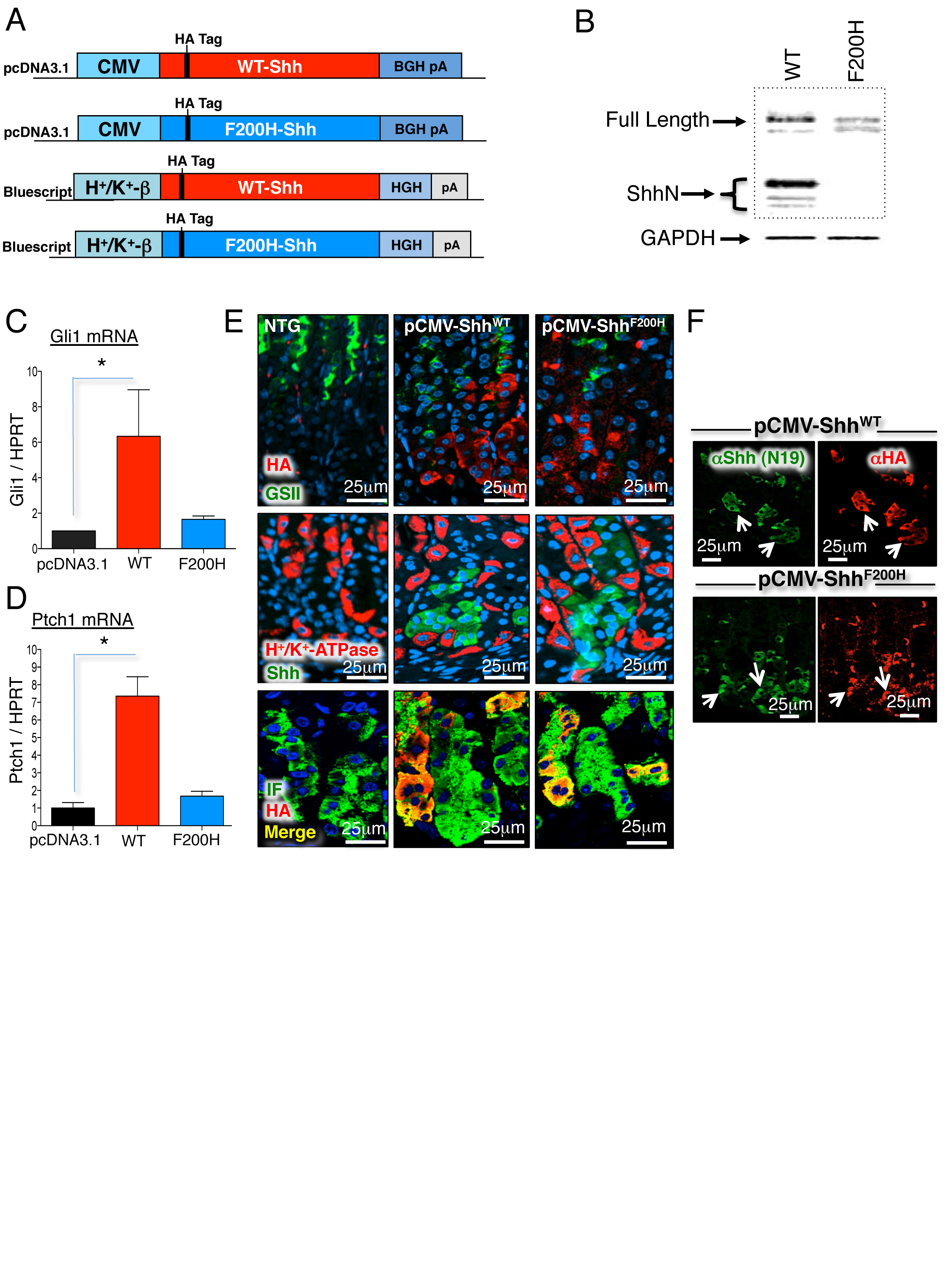

Supplement: Figure S10 — Transgene expression of Shh ligand in mice. A) Diagrammatic representation of pCMV-ShhWT, pCMV-ShhF200H, H+/K+-ATPase-β-ShhWT, and H+/K+-ATPase-β-ShhF200H constructs. The HA tag is indicated. B) Western blotting analysis of NIH-3T3 conditioned media following transfection with pCMV-ShhWT and pCMV-ShhF200H. C–D) RT-qPCR of Gli1 and Ptch1 mRNA expression in NIH-3T3 cells transfected with pCMV-ShhWT or pCMV-ShhF200H plasmids. (E) Upper Panel: Immunofluorescent staining of GSII (green) and HA-tag (red) in NTG, pCMV-ShhWT-HA, and pCMV-ShhF200H-HA mouse stomachs. Middle Panel: Shh (green) and H+/K+-ATPase (red). Lower Panel: intrinsic factor (IF; green) and HA-tag (red). (F) Immunofluorescent staining of Shh (green) and HA-tag (red) in pCMV-ShhWT and pCMV-ShhF200H mouse stomachs. A short 15 min incubation with Shh antibody was used to detect transgenes overexpressing ShhWT and ShhF200H without cross-reacting with endogenous gastric Shh. Error bars represent the mean +/− SEM. *p<0.05. (TIF) [file pone.0058935.s010.tif]

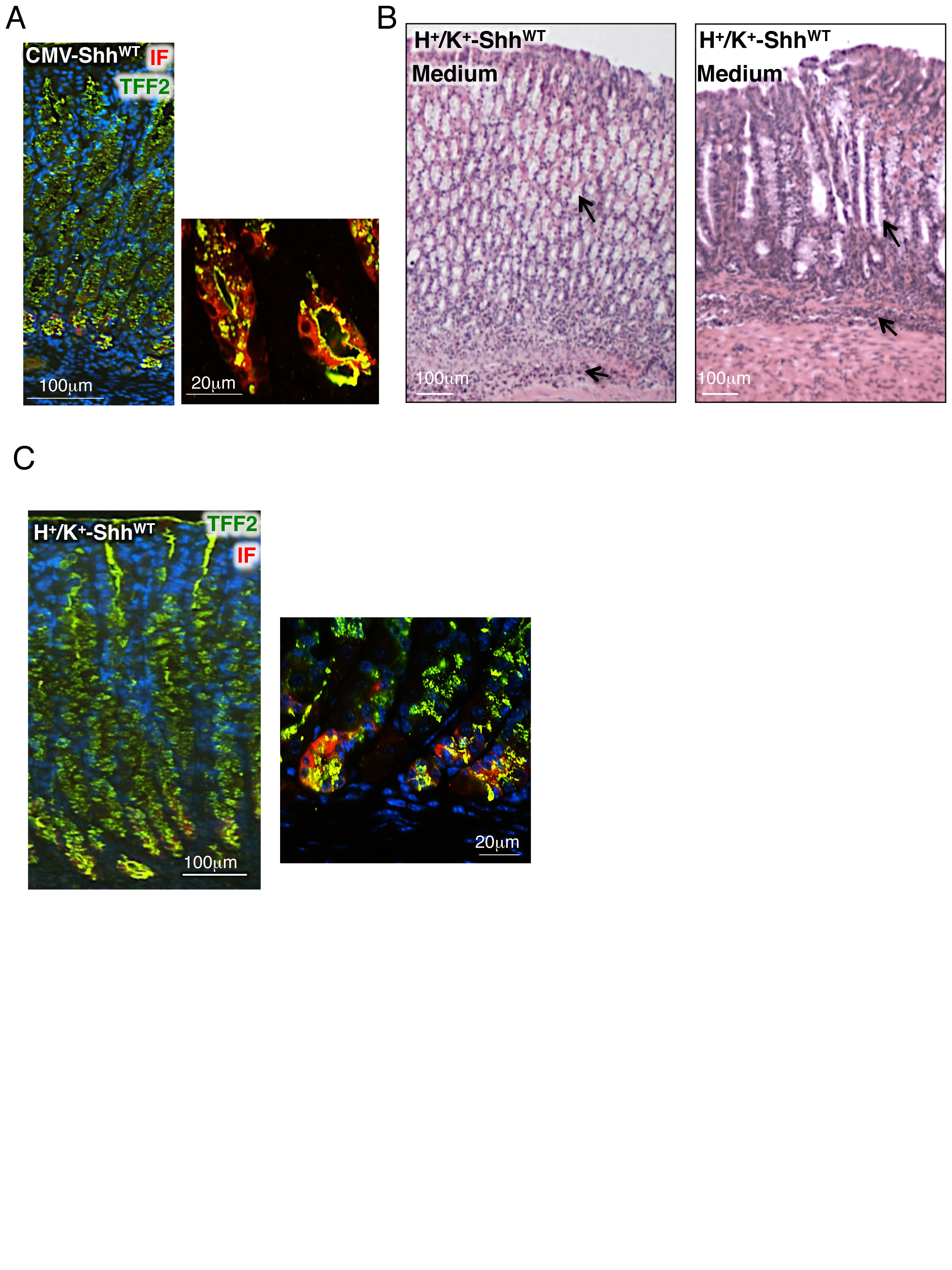

Supplement: Figure S11 — SPEM in pCMV-ShhWT and H+/K+-ATPase-β-ShhWT mice. A) Left panel, Immunofluorescent staining of TFF-2 (green), intrinsic factor (IF, red), and DAPI (blue) in the highest expressing pCMV-ShhWT mouse stomach (102-fold expression of Shh, please refer to Table 1); right panel, high power magnification of the co-localization of IF (red) and TFF-2 (green). B) H&E staining of mouse stomach in two H+/K+-ATPase-β-ShhWT founder mice expressing medium levels of the transgene (please refer to Table 1). C) Left panel, immunofluorescent staining of TFF-2 (green), intrinsic factor (IF, red), and DAPI (blue) in an H+/K+-ATPase-β-ShhWT founder mouse expressing medium levels of the transgene (please refer to Table 1); right panel, high power magnification of the co-localization of IF (red) and TFF-2 (green). Arrows indicate regions of inflammatory cell infiltration and mucous neck cell expansion. (TIF) [file pone.0058935.s011.tif]
